# Phage and Endolysin Therapy Against Antibiotics Resistant Bacteria: From Bench to Bedside

**DOI:** 10.1002/mco2.70280

**Published:** 2025-07-13

**Authors:** Majid Taati Moghadam, Samane Mohebi, Raheleh Sheikhi, Meysam Hasannejad‐Bibalan, Shahla Shahbazi, Shadman Nemati

**Affiliations:** ^1^ Department of Microbiology Virology and Microbial toxins School of Medicine Guilan University of Medical Sciences Rasht Iran; ^2^ Department of Bacteriology and Virology School of Medicine Shiraz University of Medical Sciences Shiraz Iran; ^3^ Infectious Diseases Research Center Health Policy and Promotion Institute Kermanshah University of Medical Sciences Kermanshah Iran; ^4^ Otorhinolaryngology Research Center School of Medicine Guilan University of Medical Sciences Rasht Iran

**Keywords:** antibiotic‐resistant infections, endolysin, engineered endolysins, engineered phages, phage

## Abstract

The rapid global spread of antibiotic‐resistant bacteria presents a growing public health crisis, threatening the efficacy of existing antimicrobial treatments. As traditional antibiotics become increasingly ineffective, alternative therapies such as bacteriophages and endolysins have gained renewed scientific and clinical interest. These biological agents, naturally derived from bacteriophage life cycles, exhibit potent and selective antibacterial activity, especially against multidrug‐resistant pathogens. Despite decades of research, the clinical translation of phage and endolysin therapies remains limited due to regulatory, delivery, and stability challenges. This review provides a comprehensive overview of the mechanisms, advantages, and limitations of both bacteriophages and endolysins, including their structure, mode of action, and interaction with bacterial hosts. Particular attention is given to combination therapies, where synergistic effects have been observed–especially in biofilm‐associated infections. We also explore the latest findings from preclinical studies, clinical trials, and compassionate‐use cases, with an emphasis on genetically engineered and synthetic variants that enhance therapeutic potential. Furthermore, we discuss manufacturing challenges, regulatory barriers, and future directions such as personalized phage therapy and engineered endolysins. By synthesizing current knowledge, this review highlights the academic and translational significance of phage and endolysin‐based approaches in combating antibiotic‐resistant infections.

## Graphical abstract

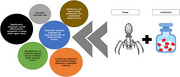

## 1 Introduction

Multidrug‐resistant (MDR) bacterial infections have emerged as one of the most pressing global health threats, currently responsible for approximately 700,000 deaths annually. Alarming forecasts from the World Health Organization project this number may rise to 10 million by 2050, surpassing cancer as a leading cause of mortality worldwide [1, 2, 3]. Although antibiotics have played a pivotal role in controlling infectious diseases since their discovery, their overuse and misuse have accelerated the emergence of resistance mechanisms, severely undermining their efficacy in clinical practice [2]. Particularly concerning are the MDR, extensively drug‐resistant (XDR), and pandrug‐resistant (PDR) strains that render all available antibiotics ineffective, challenging the management of even routine infections [4]. Among the most notorious antibiotic‐resistant pathogens are the so‐called ESKAPE organisms—*Enterococcus faecium*, *Staphylococcus aureus*, *Klebsiella pneumoniae*, *Acinetobacter baumannii*, *Pseudomonas aeruginosa*, and *Enterobacter* spp.—which are frequently associated with hospital‐acquired infections, extended hospitalizations, and significant healthcare burdens [5, 6]. The persistence of these pathogens is exacerbated by their ability to form biofilms, complex bacterial communities embedded in an exopolysaccharide matrix that confers remarkable protection from antibiotics and host immune responses. Within these biofilms, bacteria exhibit resistance levels 10 to 1000 times greater than their planktonic counterparts [7, 8]. The widespread exchange of resistance genes within these microenvironments further accelerates the proliferation of resistance traits across bacterial populations. Given the limited success of traditional antibiotics and anti‐biofilm strategies, the development of novel antimicrobial approaches has become a research priority.

One such approach is the use of bacteriophages—viruses that specifically infect bacteria—which have re‐emerged as promising therapeutic tools due to their host specificity, self‐replicating nature, and effectiveness against antibiotic‐resistant bacteria [9, 10, 11, 12]. Phages, which occur ubiquitously in nature, maintain potency where antibiotics fail and are capable of amplifying locally at infection sites without harming eukaryotic cells. Recent studies underscore their benefits, including minimal side effects, high specificity, and activity under varied physiological conditions [13, 14]. In parallel, phage‐derived enzymes known as endolysins have gained attention for their ability to degrade bacterial cell walls with remarkable precision. Endolysins are especially potent against Gram‐positive bacteria and have demonstrated synergistic effects when used in conjunction with phages or conventional antibiotics [15, 16, 17, 18, 19].

In light of these promising findings, this review examines the growing body of literature surrounding phages and endolysins as potential solutions to the antibiotic resistance crisis. It explores their mechanisms of action, therapeutic applications, limitations, and innovations such as engineered variants and combination therapies. The review also critically evaluates preclinical and clinical evidence to identify challenges in their translational use, aiming to offer a balanced and comprehensive perspective on their role in future antimicrobial strategies.

## 2 Bacteriophages: Mechanism and Therapeutic Potential

Bacteriophages, or phages, are viruses that selectively infect and destroy bacteria, making them a promising therapeutic option for bacterial infections. Phage therapy works through the precise binding of phages to target bacteria, leading to rapid bacterial lysis. Lytic phages exhibit several properties, including nontoxicity, lack of cross‐resistance with antibiotics, high specificity, bactericidal efficiency, and the ability to multiply in the presence of resistant pathogens. Unlike broad‐spectrum antibiotics, phages specifically target pathogenic bacteria without harming the beneficial microbiota, which is crucial for patients with immunodeficiency [15, 20]. Phages exert their effects on Gram‐negative and Gram‐positive antibiotic‐resistant bacteria by targeting critical processes, including peptidoglycan (PG) synthesis, cellular motility, metabolism, transcription, translation, RNA breakdown, DNA regulation, and utilizing CRISPR‐based immune mechanisms [4, 21]. Phages demonstrate effectiveness against antibiotic‐resistant biofilms by breaking down the extracellular matrix, inhibiting quorum sensing, and facilitating antibiotic penetration into the biofilm's deeper layers, making them an attractive option for innovative therapeutic approaches [4]. Based on these characteristics, phage therapy appears to hold the most promise as a future alternative to antibiotics for combating infectious diseases.

### 2.1 Bacteriophage Structure and Lifecycle

Phages coexist with their bacterial hosts, influencing various biological processes that drive microbial diversity and bacterial evolution. They possess distinct biological traits, such as morphology, optimal growth conditions, and replication dynamics. Structurally simple, phages typically consist of a core surrounded by a protein capsid. During their replication cycle, lytic phages infect bacterial cells, ultimately causing host cell lysis and the release of new phage particles [14, 20]. Originally, phages were categorized into four main morphological groups: tailed (order *Caudovirales*), polyhedral (*Microviridae*), filamentous (*Inoviridae*), and pleomorphic (*Plasmaviridae*) (15 and 16 from 3). However, a recent classification by the International Committee on Taxonomy of Viruses has expanded this taxonomy to include one class, seven orders, 31 families, 214 genera, and 858 species [22]. Phage genomes can consist of single‐stranded DNA (ssDNA), double‐stranded DNA (dsDNA), dsRNA, or ssRNA [23]. Bacteriophages are capable of specifically targeting bacteria, either by integrating into their genome as prophages or by replicating and causing bacterial lysis [12]. Bacteriophages are categorized into two groups based on their infection mechanisms: lytic and temperate. Lytic phages attach to specific host bacteria, inject their genetic material, replicate within the host, and ultimately lyse the bacterial cell, releasing progeny phages to infect nearby bacteria. In contrast, temperate phages incorporate their genetic material into the bacterial genome as prophages or persist as plasmid‐like molecules. Under favorable conditions, these prophages can activate and transition to the lytic cycle, also referred to as the lysogenic cycle [12, 20]. One of the primary limitations of using temperate phages in phage therapy is their potential to facilitate horizontal gene transfer via transduction, particularly specialized transduction mediated by temperate phages. Furthermore, temperate phages carrying virulent genes can induce lysogenic conversion, transforming nonvirulent bacteria into virulent strains, as seen in *Escherichia coli* O157:H7, which acquires prophages encoding Shiga toxin, or *Vibrio cholerae*, which gains cholera toxin via CTXΦ [24, 25, 26, 27, 28]. Therefore, the use of obligately lytic phages in phage therapy ensures bacterial eradication while minimizing the potential for antibiotic resistance through horizontal gene transfer [29].

### 2.2 Mechanism of Phage Action Against Bacteria

Phage host range, the capacity to infect various hosts, is a dynamic characteristic influenced by the density, diversity, and suitability of available host bacteria [30]. It is estimated that approximately 10^31^ phages exist globally, thriving in any environment where bacteria are present, including animal and human intestines. Despite the immense diversity of bacteriophages, half of the complete phage genomes currently available in the NCBI database focus on just seven bacterial genera: *Salmonella*, *Mycobacterium*, *Gordonia*, *Escherichia*, *Streptococcus*, *Pseudomonas*, and *Lactococcus*. This suggests that numerous phages and phage families remain undiscovered [31]. Phages are generally strain‐specific; however, certain phages can target multiple strains within a species, while others are capable of infecting strains or members across different genera [32, 33]. Phage specificity varies significantly, as seen with *Rhizobium etli* phage ph09, which has a narrow host range and infects only 4 strains within its species, compared with *S. aureus* phage ϕ812, with a broad host range capable of infecting 743 strains, including 38 coagulase‐negative *Staphylococcus* species [19]. Bacteriophages can modify their host specificity by altering their receptor‐binding proteins (RBPs). Some phages also encode multiple RBPs, enabling them to switch between different bacterial receptors [34, 35, 36].

The host range of bacteriophages is closely tied to interactions between bacterial receptors and phage tail proteins. Phage adsorption to a host cell begins with interactions between phage binding proteins and host surface receptors, facilitated by processes like Brownian motion or flow. Initially, reversible binding occurs, allowing the phage to detach and potentially infect another cell. Once primary receptor binding is stable, irreversible attachment triggers conformational changes in the phage tail machinery, leading to genome injection. Outer membrane proteins frequently serve as targets for RBPs during this process [37]. The attachment process of phages differs between Gram‐positive and Gram‐negative bacteria due to structural variations in their cell envelopes. Gram‐positive bacteria are vulnerable to phage binding through their thick PG layers, cell wall teichoic acids, lipoteichoic acids, and flagella. In contrast, Gram‐negative bacteria rely on lipopolysaccharides, pili, and capsules as primary phage attachment targets [19]. Monovalent phages target a single receptor, while polyvalent phages can bind to multiple receptors. This variability in receptor affinity contributes to the distinction between bacteriophages with a narrow host range, which infect only specific bacterial strains, and those with a broad host range, capable of infecting various strains within the same species or across different species [38, 39, 40].

### 2.3 Advantages of Phage Therapy

Phage therapy presents several advantages that surpass those of conventional antibiotics, as bacteriophages naturally target both Gram‐positive and Gram‐negative bacteria with high specificity and efficacy [41, 42]. Phages are abundantly found in diverse ecological niches, including soil, water, sewage, hospital effluents, hot springs, fecal material, and the gastrointestinal tracts of humans and animals, making their isolation cost effective compared with antibiotics. Moreover, specific phages are likely present in environments harboring particular pathogens [43]. Although it is challenging to fully assess the side effects and potential impacts of phages, they are generally considered safe, likely due to the natural daily interactions between humans and phages, which may explain the lack of reported adverse effects in humans [14]. A key feature of phages is their high host specificity, typically targeting bacteria at the species or strain level, which helps preserve the normal microbial community‐unlike antibiotics that disrupt normal flora, potentially causing super‐infections and other complications [44]. When bacteria develop resistance to one phage, they may still be susceptible to others targeting different cell surface receptors, such as lipopolysaccharides, proteins, or teichoic acids. Therefore, phage cocktails offer advantages, including greater efficacy against target bacteria and a reduced likelihood of resistance, as multiple phages can attack the same bacterial species and strains using diverse mechanisms [37, 44]. Phage therapy has shown potential in modulating the inflammatory response to infections by reducing mean C‐reactive protein levels, leukocyte counts, and possibly erythrocyte sedimentation rates, making this anti‐inflammatory effect one of its most promising aspects [45]. Phages naturally self‐replicate at infection sites, increasing their concentration and preventing the overgrowth of secondary pathogens. This reduces the need for repeated dosing, enhances treatment efficiency, and improves overall therapeutic outcomes in combating infectious diseases [14]. Bacteriophages play a crucial role in shaping bacterial communities within ecosystems, contributing to 20–40% of bacterial lysis events. Their evolution is closely tied to the density and diversity of bacterial populations in natural environments [30, 46, 47]. Broadening their host range enables phages to infect diverse hosts, but this adaptability may come with ecological trade‐offs, such as slower replication in new hosts, and evolutionary drawbacks, like diminished efficiency in their original hosts. Conversely, phages may narrow their host range in environments rich in optimal host bacteria [48, 49]. The evolution of phage host range largely depends on acquiring the ability to bind to new receptors, often through mutations in tail protein genes, as hosts with varied receptors promote the emergence of diverse phage genotypes, each adapted to specific host targets [50, 51]. Among phages targeting the same bacterial genus, genetic similarity at the amino acid sequence level remains minimal, highlighting their vast genetic diversity [31, 52, 53]. Variations in bacteriophage genomes, though still not entirely clear, significantly impact their life cycles (lytic or lysogenic) and determine the specificity and functionality of RBPs required for infecting host bacteria [20].

### 2.4 Challenges and Limitations of Phage Therapy

Different phages have demonstrated promising inhibitory effects on MDR bacteria; however, their clinical application remains limited due to several challenges. Phage therapy faces numerous challenges, including the potential transfer of antibiotic‐resistance genes between bacteria, the lack of standardized protocols for administering phage cocktails, the possibility of the immune system identifying and neutralizing phages as foreign invaders, limited genomic knowledge for many phages, rapid bacterial lysis releasing endotoxins and superantigens that may trigger inflammatory responses, the high specificity of phages complicating treatment preparation for diverse bacterial strains, the dependency on the presence of target bacteria for phage replication, limited recognition of phages as therapeutic agents, and the absence of public health insurance coverage for phage‐based treatments [14, 54]. This section outlines the key obstacles hindering the widespread adoption of phage therapy, emphasizing the need for future research to address these issues and promote its integration into clinical practice. It is widely recognized that most phages exhibit a narrow host range, typically targeting a specific natural host. However, combining multiple phages into a cocktail enhances treatment efficacy, enabling them to combat single or multiple bacterial strains effectively [55, 56]. Studies have also reported occurrences of resistance to phage cocktails; however, the molecular mechanisms driving this resistance remain unexplored. The effectiveness of commercial phage cocktails against MDR bacteria appears limited, likely due to the absence of phages targeting emerging MDR strains prevalent in various environments. To address this, specific phages targeting these contemporary MDR bacteria must be isolated, thoroughly characterized, and incorporated into existing biopreparations [57, 58, 59]. While phages are generally effective against antibiotic‐resistant bacteria, some MDR bacteria have developed defense mechanisms to resist phage infections, differing from antibiotic resistance strategies [60]. Bacteria can develop resistance to phages through mechanisms such as receptor modifications, abortive infection systems that blocking phage multiplication, restriction–modification systems; which can cleave phage DNA, development of adaptive immunity by interfering CRISPR sequences leads to the degradation of injected phage DNA, and bacteriophage exclusion and quorum sensing defense, while these mechanisms differ from those underlying antibiotic resistance, ultimately rendering phage therapy less effective, similar to antibiotic resistance [14, 60, 61]. Phage receptors can undergo phase variation or be concealed by protective barriers like capsules or extracellular polymers. These structures enhance bacterial survival in challenging environments by shielding them from adverse conditions and obstructing phages from accessing their receptors [62]. While phage cocktails provide a potential solution, there are still concerns about bacterial adaptation to multiple phages within the cocktail [14, 63]. Although phages are generally safe for eukaryotic cells, the immune system may recognize them as foreign, leading to the production of antibodies. High doses of phages should be carefully managed to avoid triggering an excessive immune response, which could accelerate their clearance from the body, reducing their therapeutic effectiveness [14, 56, 64]. Some bacterial pathogens evade phage therapy by residing within eukaryotic cells, making their receptors inaccessible to phages. Although certain phages might have the potential to enter host cells and combat intracellular bacteria, this capability is strain‐specific and requires further investigation to fully understand their interaction and therapeutic potential [56, 65, 66].

## 3 Endolysins: Mechanisms and Application

This section reviews the structure and function of endolysins, explaining how they break down bacterial cell walls to kill bacteria. It covers their effectiveness against different types of bacteria, the benefits of using them as antibacterial agents, and the challenges faced in developing endolysin‐based therapies for clinical use.

### 3.1 Structure and Function of Endolysins

#### 3.1.1 Enzymatic Degradation of Bacterial Cell Walls

Endolysins are proteins that are synthesized during the final phase of the lytic cycle of bacteriophages. Following the completion of this cycle within the host organism, endolysins act to dismantle the host's cell wall by hydrolyzing the PG, which allows for the liberation of new virions [67]. Gram‐positive bacteriophage endolysins exhibit a modular architecture, characterized by the presence of enzymatic activity domains (EADs) at the N‐terminus and cell wall binding domains (CWBDs) at the C‐terminus, which are linked by a flexible region referred to as the linker. The functionality of endolysins in enzymatic hydrolysis and substrate recognition is attributed to the EADs and CWBDs. Generally, these modular endolysins contain one or two EADs at the N‐terminal end and a CWBD at the C‐terminal end, interconnected by the linker [68]. The N‐terminal EAD is responsible for cleaving specific PG bonds within the murein layer of the host bacterium, whereas the C‐terminal CWBDs recognizes and attaches to various epitopes in the cell wall, facilitating the effective catalytic activity of the EAD [69]. Endolysins derived from bacteriophages that target Gram‐negative bacteria can exhibit various structural configurations. However, the majority possess a fundamental globular EAD domain that lacks a CWBD. Recent research has also identified Gram‐negative endolysins characterized by globular forms, featuring one or two CWBDs at the N‐terminus and the EAD module located at the C‐terminal region [70]. The classification of endolysins is determined by their cleavage sites. This category of enzymes includes lysozymes (N‐acetylmuramidases), glycosidases (N‐acetyl‐β‐d‐glucosamidases), N‐acetylmuramoyl‐l‐alanine amidases, and L‐alanoyl‐d‐glutamate endopeptidases [71]. Endolysins, which are also termed bacteriophage‐encoded cell wall hydrolases, are made up of one of four N‐terminal regions associated with a specialized cell wall‐binding domain [72].

##### 3.1.1.1 Glycosidases

The polymeric configurations of N‐acetylmuramic acid (MurNAc) and N‐acetylglucosamine (GlcNAc) are interconnected through −1,4 glycosidic bonds, which are cleaved by glycosidases. N‐acetyl‐d‐muramidase specifically hydrolyzes the bonds between GlcNAc and MurNAc units, while N‐acetyl‐d‐glucosidases target and cleave the bonds within these same residues [73]. Transglycosylases, like the other two glycosidases, target the −1,4 bonds in GlcNAc and MurNAc. In addition to this function, they are involved in an intramolecular mechanism that facilitates the creation of a 1,6‐anhydro ring at the MurNAc residue [74]. Lysozymes, or N‐acetylmuramidases, function to destroy bacteria through a targeted hydrolysis mechanism. Glycosidases, which are also known as N‐acetyl‐β‐d‐glucosamidases, regulate the hydrolysis of glycosidic bonds. The β‐1,4 glycosidic linkages serve to bind the monomers N‐acetylglucosamine (NAG) and N‐acetylmuramic acid (NAM) in PG polymers. By hydrolyzing these bonds, lysozyme compromises the structural integrity of the PG cell wall, resulting in a disruption of turgor pressure that leads to the death of the bacterial cell [75].

##### 3.1.1.2 Amidases

N‐acetylmuramoyl‐l‐alanine amidases, also known as PG amidases, operate by severing the amide bond that links the glycan strand to the stem peptide, which is found between the l‐alanine and N‐acetylmuramic acid residues [76].

##### 3.1.1.3 Endopeptidases

Endopeptidases are a class of enzymes that sever the bonds connecting amino acids in the stem peptide. l‐Alanoyl‐d‐glutamate endopeptidases, along with interpeptide bridge‐specific endopeptidases, focus on the peptide that forms the l‐alanoyl‐d‐glutamate linkage. These enzymes are capable of breaking bonds either between stem peptides or within the interpeptide bridge [67].

### 3.2 Mechanism of Bacterial Lysis by Endolysins: Activity Against Gram‐Positive and Gram‐Negative Bacteria

In the previous decade, extensive research has confirmed that the application of recombinant purified endolysin to Gram‐positive bacteria that are susceptible, including species like *Staphylococcus*, *Streptococcus*, and *Bacillus*, results in bacterial cell lysis. This phenomenon has been documented in both in vitro experiments and in several animal models simulating human diseases [77, 78]. The swift response, elevated specificity, nontoxic nature, remarkable efficacy, and minimal likelihood of resistance emergence render them as promising and innovative enzyme‐based antibacterial agents [79]. The utilization of endolysins as antibacterial agents against significant Gram‐negative pathogens is obstructed by the presence of the outer membrane. While certain endolysins exhibit some capacity to destabilize membranes, this outer membrane serves as a formidable barrier [80, 81], this outer membrane serves as a formidable barrier, effectively preventing the entry of detrimental substances, such as endolysins and various other antibacterial agents [82]. Most endolysins that act against Gram‐positive bacteria typically have a molecular weight ranging from 25 to 40 kDa and demonstrate amidase activity. They consist of sequential domains, each serving a specialized function that influences their antibacterial properties. These domains include at least one EAD at the N‐terminus and a CWBD at the C‐terminus, linked by a short, flexible connector. The CWBD identifies and binds to specific substrates on the bacterial cell wall, a feature crucial in precision medicine as it ensures selective targeting of particular bacterial strains without affecting others. Meanwhile, the EAD facilitates cell wall degradation by cleaving specific PG bonds [83]. Certain endolysins have been found to possess multiple s CWBDs or EADs organized in different sequences [84]. The exceptional modular configuration of endolysin allows for alterations and engineering innovations. The amalgamation of functional modules derived from diverse sources can change the lysis spectrum and influence its activity [85, 86]. The outer membrane (OM) acts as a defensive shield for Gram‐negative bacteria, safeguarding them against external assaults from endolysins. Consequently, the majority of endolysins that target these bacteria do not necessitate substrate recognition through carbohydrate‐binding domains (CBDs). Rather, these molecules exhibit significant diversity, typically presenting a globular form devoid of modular elements. They possess a relatively low molecular weight, typically between 15 and 20 kDa, and are characterized by a singular EAD that facilitates the hydrolysis of PG [67]. While uncommon, certain endolysins exhibit a modular architecture, including the two‐domain endolysin KZ144 from *Pseudomonas* phage [70], endolysin OBPgp279 from *P. fluorescens* phage OBP [81], and endolysin AP3gp15 from *Burkholderia* phage AP3 [87]. In contrast to those aimed at Gram‐positive bacteria, the CBD is situated at the N‐terminal, whereas the EAD is positioned at the C‐terminal [88]. The endolysin PlyC derived from streptococcal phage C1 is notable for its unique multimeric structure. This complex consists of subunits PlyCA and PlyCB, which are present in a ratio of 1:8 [89]. Worldwide initiatives to isolate and characterize phages have significantly expanded phage resources. In recent decades, phages have gained attention as a potential alternative to antimicrobial agents. With the rapid growth of phage genome databases, a limited number of naturally occurring endolysins with extracellular antimicrobial activity against Gram‐negative bacteria have been gradually identified and reported. Some of these findings were previously summarized in studies conducted before 2020 [90]. Table 1 provides a summary of newly identified endolysins and their characteristics over the past years. In general, these endolysins carry a strong positive charge at their C‐terminal regions and/or contain one or more amphipathic helices [91, 92]. There are cases where these C‐terminal segments have been proven to exhibit bactericidal effects when created as synthetic peptides [93, 94].

**TABLE 1:** Endolysins with antimicrobial activity against Gram‐negative species.

Endolysin	Key features	Target pathogens	Effective dose	In vitro performance	In vivo findings	References
PlyKp104	Contains a C‐terminal amphipathic α‐helix with positively charged residues	*K. pneumoniae, P. aeruginosa, A. baumannii, E. coli*	25 µg/mL	‐ >5 log reduction in bacterial load ‐ Stable in lung surfactant (25%), urea (500 mM), and a wide pH range (5–10)	>2 log reduction in bacterial load in skin wounds	[95]
eLysMK34	Features a cecropin A fusion at the N‐terminal via a (Ala‐Gly)₃ linker	*A. baumannii* (including colistin‐resistant strains)	0.45–1.2 µM	‐ ≥1 log reduction in stationary phase cells ‐ Effective in human serum ‐ Noncytotoxic to human cells	Not reported	[96]
Abp013	N‐terminal glycoside hydrolase (GH108) domain + C‐terminal peptidoglycan‐binding domain (9+ residues)	*A. baumannii, K. pneumoniae*	25 µg/mL	‐ >90% reduction in CFU ‐ Biofilm disruption at high concentrations	Not reported	[97]
Lysqdvp001‐ 15aa	C‐terminal fusion of a 15‐amino acid cationic peptide	*V. parahaemolyticus*	0.2 mg/mL	‐ 3.2–4.3 log reduction in 2 h ‐ Stable in 10% human serum	2.74–3.57 log reduction in oyster meat during 4°C storage	[98, 99]
PlyEc2	Glycosyl hydrolase family 19 (GH19) member with a high isoelectric point (pI 10.4)	*E. coli O157:H7, Salmonella, Shigella*	12.5–25 µg/mL	‐ NaCl‐sensitive ‐ Effective at pH 7–8.5	99.7% reduction of STEC in lettuce	[100]
P88	Engineered variant of P87 with increased net charge and hydrophobic moment	*P. aeruginosa*	5–10 µM	‐ 4–6 log reduction in 30 min ‐ Biofilm disruption ‐ Synergistic with antibiotics	Synergy with azithromycin in a pneumonia model (≈1 log reduction)	[101, 102]
Pae87	Catalytic domain with essential glutamic acids + C‐terminal antimicrobial peptide‐like region	*P. aeruginosa, M. catarrhalis*	10 mM	‐ pH‐dependent activity ‐ Sorbitol‐sensitive	Not reported	[101, 102]
BSN314	Dual enzymatic activity: N‐acetylmuramoyl‐l‐alanine amidase and d‐alanyl‐d‐alanine carboxypeptidase	*B. cereus, B. subtilis, P. aeruginosa*	1.25–2.75 µg/mL	‐ Superior to ciprofloxacin against *Bacillus* spp.	Not reported	[103]
PlyYouna2	N‐terminal amidase domain + C‐terminal DUF5776 domain	*E. coli, Yersinia enterocolitica*	1.6 µM	‐ Broad pH (7–10) and temperature (4–60°C) tolerance	Not reported	[104]
PLysChT04	Mixed α/β/coil secondary structures	*A. baumannii, E. coli, S. aureus*	312.5–625 µg/mL	Synergistic effect with colistin	Not reported	[105]
LysP53	N‐terminal 33‐aa cationic antimicrobial peptide + peptidase catalytic domain	*A. baumannii*, *P. aeruginosa*, *K. pneumoniae*, *E. coli*	100 µg/mL (in vitro), 14 µg/mouse (in vivo)	>5‐log reduction in *A. baumannii* CFU in 1 h	3‐log reduction in murine burn infection model; more effective than minocycline	[106]

Abbreviations: µg/mL, micrograms per milliliter; aa, amino acid; a. baumannii, acinetobacter baumannii; cfu, colony‐forming units; k. pneumoniae, klebsiella pneumoniae; m. catarrhalis, moraxella catarrhalis; mM, millimolar; p. aeruginosa, pseudomonas aeruginosa; pI, isoelectric point; stec, shiga toxin‐producing Escherichia coli; V. parahaemolyticus, vibrio parahaemolyticus

### 3.3 Benefits of Endolysin Therapy ‐ High Specificity and No Development of Resistance in Target Bacteria

Endolysins are characterized by their strong specificity for bacterial organisms, which allows them to avoid causing harm to human or animal cells. In contrast to antibiotics, endolysins do not share many of the beneficial traits that make antibiotics effective, such as the potential for resistance and enhanced specificity [67, 68]. Endolysins from bacteriophages generally demonstrate a strong specificity for particular species, which is regarded as a significant advantage in the current context of widespread antibiotic resistance, as it mitigates selective pressure on beneficial microbiota. The likelihood of bacteria developing resistance to endolysins is low for several reasons. First, these enzymes have evolved to target and cleave highly conserved structures in the cell walls of pathogenic bacteria. Second, the coevolution between bacteriophages and their bacterial hosts diminishes the chances of resistance to endolysins. Furthermore, because endolysins are applied externally and act on the cell wall rather than entering the bacterial cell, they bypass many resistance mechanisms (such as active efflux or reduced membrane permeability) that are common with traditional antibiotics. Additionally, many endolysins feature two catalytic domains that hydrolyze different bonds in PG, which is believed to further limit the potential for resistance development [107].

### 3.4 Challenges in Developing Endolysins for Clinical Use

#### 3.4.1 Stability and Delivery Issues

Ensuring the stability of endolysins throughout production, storage, and administration is crucial for their potential application as antibacterial agents. Preclinical studies have identified several stability challenges associated with Gram‐negative endolysins, which need to be addressed for further research and development [90]. The oral route is the most common method for delivering gut‐targeted medications and is also the preferred choice among patients. Most commercially available drugs are designed for oral use, typically in tablet or liquid form. However, this route presents certain challenges. As these medications travel through the stomach and digestive tract, they are immediately exposed to various enzymes, fluctuating pH levels, and mechanical digestion, all of which can affect their molecular stability and overall bioavailability [72]. Since endolysins are protein‐based, they are highly susceptible to degradation through these processes, which can render them ineffective. The acidic environment of the stomach can disrupt the structural integrity of certain endolysins. Additionally, proteolytic enzymes like trypsin, chymotrypsin, pepsin, and peptidase break down proteins, and many endolysins possess cleavage sites that make them vulnerable to degradation by these enzymes [108].

#### 3.4.2 Inactivation of Endolysin in Physiological Conditions

Environmental factors, such as salts and proteins, are known to significantly influence the activity of Gram‐negative endolysins. Khan et al. [109] reported that the A. baumannii endolysin LysAB54 completely loses its antibacterial effectiveness in serum, restricting its application to topical infections like burn wounds or joint infections. One possible reason for this reduction in LysAB54 activity is ion exchange, which may neutralize its antibacterial properties [109]. The inactivation of endolysin in human serum could also be due to the binding and passivation of its positively charged peptides by negatively charged serum molecules. This neutralization of the cationic domain may lead to a loss of its natural OM permeabilizing activity [110].

#### 3.4.3 Thermostability

Due to their protein‐based nature, endolysins are sensitive to temperature changes. The impact of temperature on endolysin activity has been widely researched, as protein thermostability plays a crucial role in both functionality and storage. Most endolysins exhibit optimal activity within the 30–40°C range, making them suitable for clinical applications [90].

#### 3.4.4 Storage Stability

For commercial viability, drugs with a moderately extended shelf life of 12 months when stored under refrigeration (2–8°C) or at room temperature (20–25°C) are preferred. Products requiring freezer storage (−20 ± 5°C) should maintain stability for at least 12 months [111]. However, data on the storage stability of Gram‐negative endolysins remain limited. The available information primarily focuses on short‐term storage, ranging from 1 week to 6 months, under different storage conditions [90].

#### 3.4.5 Immunogenicity Concerns

The considerations of safety, toxicity, and immunogenicity are critical during the preclinical development of protein‐based therapeutics, including endolysins. The immune system's reaction to foreign proteins, which may involve the generation of anti‐drug antibodies, can significantly impact pharmacokinetics, diminish therapeutic effectiveness, and potentially result in severe adverse effects such as hypersensitivity reactions and anaphylaxis. Reports have documented immune responses in various organisms to well‐studied endolysins like CF‐301 and SAL200 [112, 113]. Recent safety studies involving the pneumococcal endolysins Cpl‐1 and Pal have demonstrated an increase in IgG levels in mice treated with these enzymes, while IgE levels remained low. This indicates a minimal risk of hypersensitivity or allergic reactions. Additionally, the studies did not reveal any adverse health effects in the mice, nor were there any increases in pro‐inflammatory cytokine levels or complement activation. These findings collectively indicate a favorable safety and toxicity profile for these endolysins [114]. As the use of protein‐based therapeutics continues to grow, research is increasingly directed toward reducing their immunogenicity. One effective strategy involves identifying and eliminating T cell epitopes, which can be achieved through both experimental and computational methods. The computational approach has been successfully implemented in lysostaphin, a PG hydrolases bacteriocin targeting *S. aureus*, resulting in deimmunized variants that maintain their bacteriolytic activity [115, 116]. Similar approaches could also be applied to endolysins. Conversely, PG hydrolases can be utilized to trigger immune responses against harmful bacteria. Raz et al. [117] developed lysibodies, which are fusion proteins combining the CBDs of lysostaphin or various endolysins with the Fc effector region of human IgG antibodies. These lysibodies bind to *S. aureus* cells, promoting opsonization, complement activation, and phagocytosis. A comparable strategy, without incorporating antibody fragments, has also been reported for a protein derived from the CBD of the endolysin PlyV12 [118].

## 4 Combination Therapies Involving Phages and Endolysins

This section provides a comprehensive analysis of combination therapies that integrate bacteriophage‐derived endolysins with antimicrobial agents to enhance antibacterial efficacy. It addresses the synergistic mechanisms underlying these approaches, supported by empirical evidence from relevant case studies demonstrating improved bacterial clearance in clinical and industrial contexts. Furthermore, the section examines the application of endolysins in biofilm‐related infections, emphasizing their capacity to disrupt biofilms, target persistent bacterial populations, and mitigate antimicrobial resistance (AMR). Collectively, these findings highlight the therapeutic potential of phage‐endolysin combinations against MDR pathogens.

### 4.1 Synergistic Potential of Endolysin‐Based Combination Strategies

Phage‐endolysin combination therapy represents a promising approach to combat antibiotic‐resistant bacterial infections. This strategy leverages the synergistic effects of phage endolysins, which degrade bacterial cell walls, and other antimicrobial agents like peptides or antibiotics. For instance, the combination of the phage endolysin Ply2660 with the antimicrobial peptide LL‐37 has shown significant bactericidal activity against *Enterococcus faecalis*, including vancomycin‐resistant strains. This combination not only enhanced bacterial lysis but also inhibited biofilm formation, which is crucial for managing severe infections caused by MDR bacteria [119].

Another example of combination therapy involves the use of endolysins with antibiotics. Studies have demonstrated that combining endolysins with antibiotics like colistin can enhance bactericidal activity. For example, the endolysin ElyA1 combined with colistin showed improved efficacy in treating lung infections in mouse models compared with colistin alone [120]. Similarly, a combination of endolysin and vancomycin was found to be more effective than vancomycin alone in treating methicillin‐resistant S. aureus (MRSA) and VRSA infections in mouse models [121]. These findings suggest that phage‐endolysin combination therapies can offer a powerful tool in the fight against antibiotic resistance. The effectiveness of combination therapies also extends to biofilm management. Engineered endolysins, such as those with hydrophobic amino acid additions, have shown enhanced activity against *A. baumannii* biofilms when used in conjunction with phages [120]. Simultaneous treatment with endolysins and phages was found to be more effective than sequential treatment in reducing biofilm CFU counts [120]. Overall, these studies highlight the potential of phage‐endolysin combination therapies to address the growing challenge of antibiotic resistance by providing targeted and synergistic antimicrobial strategies.

### 4.2 Case Studies Demonstrating Enhanced Bacterial Clearance

1. *Treatment of Chronic Bacterial Prostatitis (CBP)*: A 39‐year‐old patient suffering from CBP caused by *E. faecalis* underwent treatment with a purified endolysin derived from the *E. faecalis* bacteriophage ϕEf11. This approach led to the elimination of the infection to below detectable levels and a substantial reduction in symptoms. Notably, the patient had previously experienced unsuccessful outcomes with multiple antibiotic courses and other therapies, underscoring the potential of endolysin therapy in managing antibiotic‐resistant infections [122].
2. *Mitigation of LactoBacillus Contamination in Corn Mash Fermentations*: In bioethanol production, bacterial contamination can hinder fermentation efficiency. The application of the recombinant endolysin LysKB317 in corn mash fermentations contaminated with *LactoBacillus fermentum* resulted in a reduction of bacterial load by approximately 3‐log units. Additionally, there was a significant decrease in lactic and acetic acid levels, leading to improved glucose utilization and a 22% increase in ethanol yield. These findings demonstrate the potential of endolysins to enhance industrial fermentation processes by controlling bacterial contaminants [123].
3. *Removal of Staphylococcal Biofilms*: Biofilms formed by *S. aureus* and *Staphylococcus epidermidis* pose challenges in clinical and food settings due to their resistance to conventional antimicrobials. The endolysin LysH5 has been shown to reduce staphylococcal sessile cell counts by 1–3 log units without inducing biofilm formation. Importantly, LysH5 effectively targeted persister cells, a subpopulation exhibiting multidrug tolerance, suggesting its potential as an adjunct to traditional antibiotics in combating biofilm‐associated infections [124].
4. *Broad‐Spectrum Activity Against MDR Bacteria*: The endolysin Abtn‐4, derived from the *A. baumannii* phage vB_AbaP_D2, exhibits broad antibacterial activity against several MDR Gram‐negative and Gram‐positive bacteria, including *A. baumannii*, *S. aureus*, *P. aeruginosa*, *K. pneumoniae*, *Enterococcus*, and *Salmonella*. In studies, Abtn‐4 reduced *A. baumannii* counts by over 3 log units within 2 h and demonstrated the ability to diminish biofilm formation. These results highlight Abtn‐4's promise as a therapeutic agent against diverse MDR bacterial infections [125].

These case studies underscore the versatility and effectiveness of endolysins in enhancing bacterial clearance across medical and industrial contexts, offering promising alternatives to traditional antimicrobial strategies.

### 4.3 Application in Biofilm‐Related Infections

Bacterial biofilms are notoriously challenging to eliminate. They develop when bacteria adhere to a surface and grow as an interconnected community of cells. This layered structure ensures that only the bacteria on the outermost layer remain susceptible to antimicrobial agents, while those embedded deeper are shielded and can continue to multiply. As a result, chronic infections often arise, necessitating prolonged antibiotic use. Over time, this treatment approach frequently leads to antibiotic resistance in biofilm‐forming bacteria. However, endolysins have recently emerged as a promising solution for tackling these persistent infections [72].

Endolysins play a crucial role in the treatment of biofilms, demonstrating notable effectiveness as evidenced by numerous studies. For instance, Guo and colleagues [126] employed the innovative endolysin LysPA26 to successfully eradicate *P. aeruginosa* during biofilm development. LysPA26 has the capability to lyse various Gram‐negative bacteria, including *A. baumannii*, *K. pneumoniae*, and *Escherichia coli*, across a wide temperature spectrum ranging from 37 to 50°C. The research findings revealed that LysPA26 effectively degrades biofilms and disrupts bacterial cells in a concentration‐dependent manner, as evidenced by a decrease in biofilm optical density (OD600) [126]. Meng et al. [127] investigated the effectiveness of the engineered bacteriophage lysin, LySMP, in treating *Streptococcus suis* biofilm, both alone and in combination with antibiotics and bacteriophage. They discovered that LySMP alone could eliminate over 80% of the biofilm, while bacteriophage treatment alone or combined with antibiotics resulted in less than 20% removal. The findings showed that LySMP could synergistically treat *S. suis* biofilm in a concentration‐independent manner and also inactivate the released cells [127].

In research utilizing animal models, the engineered PG hydrolase has been applied to cleave significant bonds in the PG structure of *S. aureus*, resulting in an increased rate of bacterial colony lysis and improved biofilm removal [128]. Recently, glycoside hydrolases have shown promise in disrupting P. aeruginosa biofilms and facilitating the eradication of neutrophil‐mediated communities [128]. Moreover, fusion proteins derived from bacteriophage‐encoded endolysins can effectively reduce the resistance of bacterial communities within biofilms while leaving commensal bacteria unharmed [129].

Persister cells are a minor subset of bacterial populations that exhibit significant resistance to antibiotics. This resistance arises from their capacity to enter a dormant phase when exposed to bactericidal antibiotics, allowing them to endure treatment [130]. Consequently, persister cells pose a significant challenge in the eradication of biofilms. Beyond their ability to resist antibiotics, they have the capacity to repopulate immediately following the alleviation of stress [131]. Gutiérrez et al. employed the phage‐derived lysin, LysH5, to eliminate persister cells of *S. aureus* in biofilms [124]. The findings indicated that no persister cells were present in the Staphylococcal biofilm after treatment with 0.15 µM of LysH5. There was also a complete inhibition in biofilm formation in select strains. These studies emphasized several advantageous characteristics of endolysins in combating biofilms, such as their capability to penetrate deep into the biofilm structure and the absence of resistance development against them.

## 5 Preclinical Studies: Efficacy in Animal Models

### 5.1 Overview of in Vitro and in Vivo Studies on Phage and Endolysin Therapy

The global rise in MDR bacterial infections poses a critical threat to public health, significantly burdening healthcare systems. In the United States alone, billions of dollars are spent annually to manage drug‐resistant infections [14]. As antibiotic resistance escalates, phage and endolysin therapies have garnered increasing attention as potential alternatives to conventional antibiotics, particularly for combating MDR bacteria. Both therapies offer promising solutions in addressing the growing AMR crisis, which threatens the effective prevention and control of infectious diseases [132, 133]. Phage therapy, which involves using bacteriophages to treat bacterial infections, has been practiced for nearly a century. This therapeutic approach primarily relies on lytic bacteriophages, as well as purified phage lytic proteins, to target and lyse bacteria directly at the site of infection [4, 29]. Current research demonstrates that phage therapy has significant potential as an alternative to antibiotics, whether phages are used independently or in combination with conventional antibiotics [29, 132]. Phage‐derived endolysins, which are cell wall hydrolases, can break down the bacterial PG layer from both within and outside bacterial cells. Endolysins are highly specific, act rapidly, and exhibit a low risk of resistance development, making them promising alternative therapies for MDR bacteria [76, 133]. Recent studies have underscored the efficacy of bacteriophage and endolysin therapy, both in vitro and in vivo. Bacteriophage‐derived endolysins have shown effectiveness against a variety of pathogens, including Gram‐positive and Gram‐negative species [90, 134]. When combined with antibiotics and/or loaded in nanoparticles these endolysins can have synergistic effects, improving therapeutic outcomes [135, 136]. Animal models have validated the therapeutic potential of endolysins, showing significant reductions in bacterial loads and improved survival rates in conditions like pneumonia, sepsis, and wound infections. These studies consistently report a favorable safety profile with minimal adverse effects (Table 2). In vitro studies also provide promising evidence of phages’ and endolysins’ efficacy against MDR bacteria. Phages have been shown to lyse MDR S. aureus, including MRSA, and achieve rapid bacterial clearance in animal models [137]. Phage cocktails targeting MDR bacteria in septic wounds have proven effective in lysing pathogens such as K. pneumoniae, S. aureus, P. aeruginosa, and E. coli [29, 138, 139]. Furthermore, several studies indicate that phages possess the ability to inhibit biofilm formation, a critical contributor to chronic infections [139]. Additionally, research has demonstrated the efficacy of phages against colistin‐resistant, MDR, and XDR Gram‐negative bacteria [4, 140, 141]. Phage therapy has also shown promising results in treating various infection models in animals (Table 2). For example, the systemic administration of phages has reduced bacterial burdens and improved survival in animal models of sepsis, osteomyelitis, intestinal infections, and pneumonia [142]. These therapies have even been applied in challenging scenarios, such as MDR wound infections, where phage cocktails have been used to promote wound healing and prevent further tissue damage. Studies have documented successful outcomes against infections caused by A. baumannii, with phage therapy halting bacterial proliferation and supporting tissue regeneration [143]. Furthermore, Indian Council of Medical Research investigations on mycobacteriophages and phage enzymes for tuberculosis treatment revealed that specific enzymes, such as D29 LysB, are effective against both susceptible and drug‐resistant strains of M. tuberculosis [132]. Overall, phage and endolysin therapies represent a valuable new approach to treating MDR, XDR, and PDR bacterial infections. The promising results from in vitro and in vivo studies indicate that these therapies can potentially serve as effective alternatives or adjuncts to traditional antibiotic treatments. With ongoing research, there is hope that phages and endolysins can fill the gap left by the declining efficacy of conventional antibiotics, offering solutions for treating severe bacterial infections across various clinical contexts.

**TABLE 2:** Overview of preclinical studies on phage and endolysin therapy in animal models.

Bacterial pathogen	Model system	Therapy type	Dose optimization	Key outcomes	Safety and toxicity	References
MRSA	Rat model of soft tissue infections	Two *Myoviridae* bacteriophages (phages MR‐5 and MR‐10), administered as FPC and TPC (i.m. administration)	In study 1, FPC and TPC were administered at MOI 10. Study 2 compared immediate (30 min) and delayed (12 h) treatments with linezolid as a control	The transfer of some‐entrapped bacteriophage cocktails saved all the infected animals.	TPC showed a favorable safety profile with no fatalities and reduced tissue damage compared with untreated controls, while FPC increased mortality, indicating higher toxicity.	[144]
MDR *A. baumannii*	Mouse bacteremia model	Phage cocktail (vB_AbaS_D0 + vB_AbaP_D2) (i.p. administration)	100 µL of phage (10⁹ PFU/mL) administered 2 h postinfection with 10×LD100 of A. baumannii	Reduced bacterial load, lower phage resistance; improved survival rates	No adverse effects noted; significant efficacy observed	[143]
Antibiotic resistant *S.aureus*	Neutropenic and immunocompetent mouse models of acute pneumonia	Phage cocktail (AB‐SA01: 3 myoviruses) (i.n. administration)	5.10^8^ PFU per phage intranasally at 2 and 6 h postinfection	Phage treatment effectively reduced bacterial load in lungs of neutropenic mice.	Phages were safe and well tolerated.	[145]
MDR* S. aureus*	Diabetic mouse wound infection model	Cocktail of *three S. aureus Myoviridae* phages (AB‐SA01) (Topical application)	70 µL AB‐SA01, equivalent to 7.9 log_10_ PFU	Phage treatment reduced bacterial load and wound size.	Phage therapy was safe and as effective as vancomycin.	[146]
MRSA	Rat model of ventilator‐associated pneumonia	2003, 2002, 3A, and K nebulized phages (i.v. administration)	Administration of 2.10^10^ PFU directly into the lungs at 2, 12, 24, 48, and 72 h postinfection	Combination showed no improvement in survival or bacterial reduction (55 vs. 50% survival).	No adverse effects noted; combination treatment lacked additional therapeutic benefit. Further optimization needed	[147]
XDR P. aeruginosa	Liquid infection model; biofilm on catheters and glass slips	Phage cocktail (φPA170, φPA172, φPA177, φPA180)	10^2^–10^7^ PFU/mL	Significant inhibition of bacterial growth, effective eradication of biofilms; optimal cocktail improved lytic range	No toxicity reported; excess phages reduced overall inhibition	[140]
MDR *P. aeruginosa*	Mouse bacteremia model)nonneutropenic BalB/C mice(	Phage therapy (Pseudomonas phage AP025 and AP006) (i.p. administration)	Administered a single dose of AP025 and AP006 at MOIs of 1 (8 × 10^6^ PFU/mice), 10 (8 × 10^7^ PFU/mice), and 100 (8 × 10^8^ PFU/mice)	Significant reduction in bacterial load; improved survival rates	No adverse effects reported	[148]
*S. aureus*	Mastitis in mice	Phage cocktail (vBSM‐A1 ‐ *Myoviridae*, vBSP‐A2 ‐ *Podoviridae*)	Phage cocktail (5 × 10⁷ PFU/gland) administered 4 h postinfection.	Phage cocktail significantly reduced bacterial counts and improved mastitis symptoms.	No toxicity or systemic spread observed, and safety was comparable to antibiotic treatment.	[149]
Ciprofoxacin/ceftriaxoneresistant *E.coli*	Galleria mellonella invertebrate infection model	ɸWL‐3	10^8 ^PFU/mL	ɸWL‐3 improved antibiotic efficacy, reducing MBBC by up to 512 times; enhanced survival in ATCC 25922‐infected larvae.	Better survival with ɸWL‐3/fosfomycin; higher endotoxin release in EC1‐infected larvae may reduce efficacy.	[150]
Vancomycin‐resistant *E. faecium*	*G. mellonella* larva	Monophage (vB_EfaH_163) (i.h. administration)	Phage solution in the second‐to‐last left proleg at MOI 0.1 for 5 days	Larval survival rate improved by 20%.	The vB_EfaH_163 phage showed no virulence factors or resistance genes and effectively controlled clinical vancomycin‐resistant E. faecium in vitro and in a G. mellonella model.	[151]
Human pathogenic *E. coli* isolates	Rat model for artificial incubation of pathogenic E. coli	Phage cocktail (140 pages)	Drinking water: 10^7^ PFU/mL for 20 days. Oral Injection: 4 mL phage suspension three times daily for 20 days. Capsules: 0.5 mL phage cocktail (10^6^ PFU/mL) in three capsules daily for 20 day	Vegetable capsules resulted in the greatest reduction of fecal E. coli, followed by the drinking water method of phage administration.	The phage cocktail was safe for white rats, with no adverse effects noted during treatment.	[152]
MDR *P. aeruginosa*	New Zealand rabbit skin infection model	PaVOA and phage cocktail (Topical application)	2 mL of 10⁸ PFU/mL phage or 0.002% ceftriaxone applied to gauze for 4 days	Phage cocktail improved healing and skin remodeling vs. ceftriaxone and phage alone.	No deaths; phage inactivated in blood after 12 h, safe for topical use.	[153]
PDR *P. aeruginosa*	Mouse systemic infection model	Monophage (PELP20), and phage cocktail (PELP20, PT6, PMN and 14/1) and antibiotic meropenem (i.n. and i.v. administration)	50 µL of phages PELP20 and PT6 or a phage cocktail at 5 × 10⁹ PFU, or 50 µL of phages PNM and 14/1 at 4.95 × 10¹^2^ PFU	IV phage cocktail delivery proved more effective than delayed IN administration, achieving complete bacterial clearance when combined with meropenem.	Strong infection clearance; phage resistance increased antibiotic sensitivity, but endotoxin removal is needed for clinical use.	[154]
*S. aureus*	Peri‐prosthetic joint infections in rats	Five *Myoviridae* bacteriophages (StaPh_1, StaPh_3, StaPh_4, StaPh_11, and StaPh_16) (i.p. administration)	StaPhage cocktail (1.3 × 10^8^ PFU; MOI, > 10^4^ PFU:1 CFU) was administered on day 21, 22, and 23 postsurgery	Phage therapy significantly reduced bacterial load, especially in combination with vancomycin, leading to decreased joint swelling and inflammation	Phage therapy was well tolerated with no significant adverse effects.	[155]
Vancomycin‐resistant *E. faecalis*	Murine septicemia model	Monophage (Phage EF‐P29) (i.p. administration)	Single dose (4 × 10^3^ to 4 × 10⁶ PFU/mouse)	EF‐P29 effectively cleared VRE in mice, with dose‐dependent reductions and restored gut microbiota balance.	EF‐P29 showed no adverse effects and lacks harmful genes. It restored gut microbiota but requires controlled dosing to avoid imbalance.	[156]
Vancomycin‐resistant *E. faecalis*	Mouse severe septic peritonitis model	Bacteriophage cocktail of EFDG1 and EFLK1 (i.p. administration)	2 × 10^8^ PFU/mL	Single phage cocktail treatment was enough to eliminate 100% of mortality; significant reduction in liver and heart bacterial loads.	No significant alterations in gut microbiome; bacteriophage therapy showed no toxic effects, while antibiotics are known to cause dysbiosis.	[157]
MDR *E. cloacae* complex	Murine septicemia model	Phage cocktail‐ V1 (3 phages øEnA02, øEnC07 and øEnC15) (i.p. administration)	A single 200 mL dose of PBS with phages at 1 × 10^8^ PFU/mL was given 1 h postinfection.	Treatment achieved >99.9% bacterial load reduction in blood, kidney, liver, and spleen, with higher phage concentrations in the liver and spleen.	Phage therapy achieved >99% bacterial load reduction in septicemic mice, with Entelli‐02 demonstrating 99% host coverage and 92% efficacy against *E. cloacae* complex.	[158]
PDR *A. baumannii*	Mouse bacteremia model	Monophage (phage ФAb4B) and antibiotic (CIP) (i.p. and i.v. administration)	Injections of 1 × 10^9^ PFU phages and ciprofloxacin (30 mg/kg) for 7 days	The phage‐CIP combo saved 91% of mice, compared with 67% for phage alone, and boosted neutrophil counts more than ciprofloxacin.	ФAb4B phage therapy significantly rescued 91% of mice and improved neutrophil counts without toxicity	[159]
MRSA	Mouse peritonitis model	LysSYL (i.p. administration)	A single 50 mg/kg dose	LysSYL rapidly lysed MRSA, eliminated biofilms, and rescued 100% of infected mice.	LysSYL was safe and fully protected mice from S. aureus infection at 50 mg/kg, matching vancomycin's efficacy.	[160]
MDR *S. aureus*	Mouse nasal MRSA infection model	ϕMR11 (MV‐L lysin) (i.p. administration)	500 U (optimal), 1500 U, 2000 U (excess doses) at 0 and 30 min postinfection.	Bacteriophage ϕMR11 efficiently lysed MDR S. aureus under growth conditions and eliminated MRSA infections in vivo in mice.	MV‐L demonstrated high efficacy in protecting mice from lethality caused by MRSA, with a 500 U dose effectively reducing bacterial load and improving survival. Higher doses did not adversely affect safety.	[161]
MRSA and MDR Gram‐negative bacteria	Mouse systemic infection model	LysSS endolysin (i.p. administration)	LysSS doses of 125 and 500 µg administered intraperitoneally. Treatment was given 30 min postinfection, and survival rates were monitored for 6 days.	LysSS effectively targets MDR pathogens with an MIC of 0.063–0.25 mg/mL and no cytotoxicity in A549 cells below 250 µg/mL. It provided a 40% survival rate in infected mice at 125 µg.	LysSS was effective against MDR bacteria with no cytotoxicity in human lung cells below 250 µg/mL and 40% survival in mice treated with 125 µg.	[91]
MDR and colistin‐resistant *A*. *baumannii*	Mouse model of bacteremia	LysAB2P3 endolysin (i.p. administration)	100 µM/mouse, 3.7 mg/kg, 1 h postinfection	Reduces bacterial burden significantly in mice infection model and prevented deadly bacteremia in 60% of infected mice.	LysAB2 P3 exhibited minimal hemolytic activity and no cytotoxicity toward eukaryotic cells.	[94]
MDR *P. aeruginosa*	Mouse models of skin Infection	PlyPa03 endolysin (Dermal)	Two sequential 25 µL doses of lysin PlyPa03 (approximately 300 µg)	The mean bacterial load decreased by 2 logs.	>5‐log killing activity against P. s aeruginosa. No activity in 100% human serum.	[162]
MDR *P. aeruginosa*	Mouse models of lung infection	PlyPa91 endolysin (i.n. and i.h. administration)	50 µL of 1.8 mg/mL PlyPa91; administered either as two intranasal instillations or as one intranasal and one intratracheal instillation	Two intranasal instillations resulted in a significant delay in mortality, achieving a 20% survival rate. In contrast, one intranasal and one intratracheal instillation led to a reduction in the death rate, with a 70% survival rate.	>5‐log killing activity against P. aeruginosa. effective in lower serum concentrations but inactive in 100% serum.	[162]
*K. pneumonia*	Intraperitoneal infection model using *K. pneumoniae* in mice	DP42 endolysin (i.p. administration)	200 µL of Dp42 (approximately 50 µg)	Prophylactic treatment with Dp42 led to a 100% survival rate in mice. Additionally, administering Dp42 after infection markedly improved the survival rates of the infected mice.	Endolysin Dp42 showed no adverse effects in healthy mice and provided 100% survival at lower infection doses (2 × 10⁷ cfu). Efficacy decreased to 25% at higher bacterial loads (2 × 10⁸ cfu)	[163]
*S. pneumoniae*	Mouse severe pneumonia model	Cpl‐1 endolysin (i.p. administration)	1 mg 24, 36, 48, 60, 72, and 84 h after infection	Cpl‐1 treatment led to 100% survival at 24 h and 42% survival at 48 h, reducing bacterial load and inflammation.	Reduces bacteria and inflammation, showing similar efficacy to amoxicillin with lower inflammatory response.	[164]
MDR *A. baumannii*	Mouse sepsis model	Monophage (phage PD‐6A3); phage cocktail (composed of 14 phages: PD‐Ab1, PD‐Ab8, PD‐Ab9, PD‐Ab11, PD‐Ab15, PD‐Ab16, PD‐Ab17, PD‐Ab18, PD‐6A1, PD‐6A2, PD‐6A3, PD‐6A4, PD‐7A1, and PD‐7Ab3); phage‐derived enzyme (Ply6A3) (i.p. administration)	1 mL of endolysin Ply6A3 at 2 mg/mL, 1 mL of phage PD‐6A3 (10⁹ PFU/mL), and 1 mL of phage cocktail (10⁹ PFU/mL)	The endolysin therapy group exhibited milder clinical symptoms compared with the other treatment group, with a 70% survival rate.	No cytotoxicity or side effects in treated cells and mice. Combination of phage PD6A3 and endolysin Ply6A3 showed superior activity.	[165]

Abbreviations: a. baumannii, acinetobacter baumannii; atcc, american type culture collection; cfu, colony‐forming unit; cip, ciprofloxacin; e. cloacae, enterobacter cloacae; e. coli, escherichia coli; e. faecalis, enterococcus faecalis; e. faecium, enterococcus faecium; fpc, first phage cocktail; g. mellonella, galleria mellonella; i.m, intramuscular; i.n, intranasal; i.p, intraperitoneal; i.v, intravenous; lys, lysin; mbbc, minimal biofilm bactericidal concentration; mdr, multidrug‐resistant; mic, minimum inhibitory concentration; moi, multiplicity of infection; mrsa, methicillin‐resistant staphylococcus aureus; p. aeruginosa, pseudomonas aeruginosa; pdr, pandrug‐resistant; pfu, plaque‐forming unit; s. aureus, staphylococcus aureus; tpc, test phage cocktail; xdr, extensively drug‐resistant.

### 5.2 Dose Optimization, Safety, and Toxicity Studies

Prior to initiating clinical trials, thorough assessment of phage safety and efficacy is essential for therapeutic application. This process involves establishing infection parameters and stability profiles in vitro, followed by testing in relevant infection models, such as biofilms or ex vivo simulations [166]. For instance, studies examining human pathogens like E. faecium and E. faecalis in an ex vivo biofilm collagen wound model have shown encouraging initial outcomes. However, issues such as bacterial regrowth and phage resistance emergence were noted posttreatment [167]. To address these challenges, strategies including phage cocktails and phage‐antibiotic synergy have been suggested. While in vitro and ex vivo studies provide foundational insights, they cannot fully ensure safety and efficacy within the complexities of the human body. Consequently, advanced testing in appropriate in vivo infection models is crucial for clinical applications. Animal models, which simulate human infections, offer critical data on phage safety, efficacy, toxicology, and pharmacokinetics. In animal studies, the use of purified phage preparations and customized delivery methods is essential to accurately assess therapeutic regimens, dosages, vital sign monitoring, bacterial colonization, histopathology, immune responses, and wound healing [132]. The choice of animal models in phage therapy studies depends on their ability to replicate human infections effectively. Various animals have been employed in preclinical trials to better understand phage efficacy, mechanisms of action, immune system interactions, and safety profiles, as summarized in Table 2. For example, a study evaluated the efficacy of phages against MDR S. aureus in diabetic mouse wound infections. The researchers developed a three‐phage cocktail under good manufacturing practices (GMP), finding that phage treatment significantly reduced bacterial load and wound size in mice, with results comparable to those from vancomycin treatment. These findings support the clinical potential of phages in treating diabetic wounds [146]. Studies using both invertebrates (e.g., wax moths [151, 168] and zebrafish [169]) and vertebrates (e.g., rabbits [170], rats [171], and mice [172]) demonstrate that animal models offer a cost effective, rapid, and ethically sound alternative to human clinical trials. Addressing these preclinical considerations is paramount for the eventual application of phage therapies in human patients [173]. Recent systematic reviews have further emphasized the safety and toxicity of phage therapy, revealing that while adverse events have been documented, serious incidents remain extremely rare. A comprehensive analysis of 69 publications, which included 20 animal studies and 14 clinical trials, highlighted the lack of standardized reporting on potential toxicities associated with phage therapy. Recommendations have been made for structured safety and tolerability endpoints, which should be integral to both clinical and preclinical studies. Notably, the optimization of safety monitoring during phage therapy is essential, with emphasis on factors such as dosing variability and the effects on pregnancy, growth, and development [174]. Despite the promising safety profile, the efficacy of phage therapy has not been uniformly demonstrated across clinical trials. A systematic review found that out of 13 modern trials, all concluded phage therapy was safe, yet only two trials reported efficacy. This discrepancy may arise from trials not adequately delivering therapeutic amounts of the appropriate phage(s) to the site of infection, particularly in patients who have already failed antibiotic therapy. Compelling clinical evidence indicates that, despite these challenges, phage therapy can effectively resolve infections resistant to conventional antibiotics [175]. Addressing these preclinical considerations and optimizing safety monitoring are paramount for the eventual application of phage therapies in human patients. Comprehensive assessments of safety will likely benefit from standardization, facilitating interstudy comparisons and paving the way for successful phage therapy implementations. Endolysins exhibit potent bactericidal activity against bacterial strains closely related to their source phages. Their host range can extend to approximately two‐thirds of tested strains, and in some cases, they have achieved 100% efficacy, which is a broader range compared with the phage itself [133]. For example, the pneumococcal phage endolysin Pal can target 15 pneumococcal serotypes, including highly penicillin‐resistant strains [176]. Moreover, endolysins like LysPBC2 show broad‐spectrum lytic activity against multiple genera, such as Bacillus, Listeria, and Clostridium [177]. The endolysin PlyV12 from enterococcal phages can effectively kill enterococci and other Gram‐positive pathogens, including streptococci and staphylococci [178]. Many endolysins demonstrate a dose‐dependent bactericidal effect. For instance, PlyG, at a dose of 2 units, can eliminate 1.0 × 10⁴ CFU of streptomycin‐resistant B. cereus in just 10 s [179]. In a separate study, PlyG achieved a 17,000‐fold reduction in B. cereus population within 20 s [179]. Thus, endolysins offer robust lytic efficacy even at low doses. However, endolysin immunogenicity poses concerns about neutralizing antibodies diminishing in vivo efficacy [180, 181]. Nonetheless, studies show that antibodies against specific endolysins (e.g., LysGH15) do not significantly impair their in vitro lytic activity [77, 182]. Additionally, immunized rabbit serum does not reduce the effectiveness of Cpl‐1, Pal, and MV‐L endolysins, supporting their potential clinical application despite immunogenicity [161, 183, 184]. Terms of safety, multiple studies affirm endolysins’ safety in animal models. For example, no histopathological abnormalities were noted in mice treated with Group A streptococcal‐specific endolysins [185]. Moreover, SAL200, targeting S. aureus, demonstrated no toxicity in preclinical tests on mice, dogs, and monkeys [186, 187, 188]. In human trials, SAL200 was well‐tolerated, with minor adverse effects like fatigue and headache that were transient and self‐limiting [186]. Similarly, LysGH15, even at high doses, showed no significant side effects or tissue pathology in mice [182]. Although endolysins appear safe due to the lack of PG in human cells, which they target, concerns remain about inflammatory responses from bacterial component release during bacteriolysis [189, 190]. Nonetheless, the absence of significant adverse events in studies such as those on SAL200 and CF‐301 supports endolysin safety for therapeutic use [181, 187, 191].

## 6 Clinical Trials and Current Applications

### 6.1 Summary of Ongoing and Completed Clinical Trials

A key milestone for any scientific field is the shift from anecdotal evidence to reproducible, measurable outcomes. Bacteriophage therapy seems to be at such a transition, as indicated by the recent surge in clinical trials (Table 3). From 2013 to 2024, a total of 41 clinical trials investigating phage therapy were conducted across various countries. These trials primarily focused on assessing the safety, efficacy, and therapeutic applications of bacteriophages in combating infections caused by MDR bacteria. A significant portion of the studies advanced to Phase II, reflecting the increasing interest in phage therapy as an alternative to conventional antibiotics. Most trials targeted bacterial pathogens such as *P. aeruginosa, S. aureus*, and *E. coli*, which are commonly associated with antibiotic resistance. In terms of clinical applications, the majority of studies addressed wound infections, respiratory tract infections, and urinary tract infections, with a smaller number focusing on systemic infections. While the majority of trials were conducted in the US and Europe, there is growing recognition of the importance of conducting phage therapy research in diverse geographic regions, including low‐ and middle‐income countries. The findings from completed trials have been encouraging, demonstrating reductions in bacterial loads and improvements in patient outcomes. However, some trials were limited by small sample sizes, pointing to the necessity for larger, more robust studies to validate these results and establish the broader clinical utility of phage therapy. Endolysin therapies are also being actively pursued in clinical research. ContraFect has acquired the rights to nine phage‐derived endolysins for the treatment of bacterial infections. One of its lead candidates, Exebacase (CF‐301), targets Streptococcus and Staphylococcus species, including MRSA. While early Phase II trials showed promising results—with a reported 42.8% improvement in recovery rates for MRSA endocarditis when used in combination with antibiotics—a subsequent Phase III trial (NCT04160468) was terminated early due to lack of efficacy observed in interim analysis [192, 193, 194]. These findings highlight both the potential and the challenges of translating endolysin therapies into clinical success. iNtRON Biopharma also completed a Phase III trial in 2021 for N‐Rephasin Sal200 (NCT03089697), showing its potential as a clinical treatment without significant side effects [186]. Despite these advances, further research is needed to optimize endolysin delivery and expand their clinical applications, particularly in large animal models and human trials

**TABLE 3:** Summary of clinical trials investigating phage and endolysin therapies conducted worldwide from 2013 to 2024.

Study	Study status	Conditions	Interventions	Sponsor	Phases	NCT number
**Phage**						
Bacteriophage therapy in spinal cord injury patients with bacteriuria	Recruiting	Bacteriuria, spinal cord injuries, asymptomatic bacteriuria, *E. coli*	Drug: phage therapy (TAILФR phage cocktail) Other: placebo (sterile 0.9% saline)	Barbara Wells Trautner	Phase 1b	NCT06559618
Proof of concept study to assess safety and efficacy of phage therapy in hip or knee PJI due to *S. aureus* treated by DAIR.	Not yet recruiting	Hip prosthesis infection Knee prosthesis infection	Biological: anti‐*S. aureus* bacteriophages (PP1493 and PP1815) intra‐articular injection with 0.9% NaCl solution Drug: 0.9% NaCl solution	Phaxiam Therapeutics	Phase 2	NCT06605651
Bacteriophage therapy in patients with PJI	Withdrawn	PJI	Drug: bacteriophage Drug: placebo	Adaptive Phage Therapeutics, Inc	Phase 2	NCT05269134
Bacteriophage therapy in first time chronic PJI	Withdrawn	PJI, bacterial infections	Phage therapy	Adaptive Phage Therapeutics, Inc.	Phase 1/2	NCT05269121
Phage therapy for MSSA prosthetic joint infection	Active, not recruiting	PJI of hip, *S. aureus* infection	Phage therapy (bacteriophage cocktail BP13 and J1P3)	University of Calgary	Phase 1/2	NCT06456424
Individual patient expanded access for AB‐PA01, an investigational anti‐*P. aeruginosa* bacteriophage therapeutic	No longer available	Serious or life‐threatening *P. aeruginosa* infections	AB‐PA01	Armata Pharmaceuticals, Inc.	Not applicable (Expanded Access)	NCT03395743
Phage therapy for recurrent UTIs in kidney transplant recipients	Not yet recruiting	UTI, recurrent	Drug: phage therapy, drug: control	University of California, San Diego	Phase 1/2	NCT06409819
Phase 1/​2a to assess the safety and tolerability of TP‐122A for the treatment of Ventilator‐Associated Pneumonia	Not yet recruiting	Pneumonia, ventilator‐associated	TP‐122A	Technophage, SA	Phase 1/2	NCT06370598
Phage therapy in prosthetic joint infection due to *S. aureus* treated with DAIR.	Recruiting	Infection of total hip joint prosthesis, Infection of total knee joint prosthesis	Anti‐*S. aureus* bacteriophages (PP1493 and PP1815)	Phaxiam Therapeutics	Phase 2	NCT05369104
Bacteriophage therapy for difficult‐to‐treat infections: the implementation of a multidisciplinary phage task force	Recruiting	Musculoskeletal infection, chronic rhinosinusitis, sepsis, pulmonary infection, hidradenitis suppurativa	Other: prospective data collection	Universitaire Ziekenhuizen KU Leuven	*	NCT06368388
Study evaluating safety, tolerability, and efficacy of intravenous AP‐SA02 in subjects with *S. aureus* bacteremia	Completed	Bacteremia; *S. aureus*; *S. aureus* bacteremia	Biological: AP‐SA02 Other: placebo	Armata Pharmaceuticals, Inc.	Phase 1/2	NCT05184764
Individual patient expanded access for AB‐SA01, an investigational anti‐*S. aureus* bacteriophage therapeutic	No longer available	Serious or life‐threatening *S. aureus* infections	AB‐SA01	Armata Pharmaceuticals, Inc.	Not applicable (Expanded Access)	NCT03395769
Clinical trial to demonstrate the safety and efficacy of DUOFAGÂ®	Recruiting	Surgical site infection, *S. aureus*, *P. aeruginosa*, bacterial infections, surgical wound infection	Drug: IMP (DUOFAG® phage cocktail) Drug: placebo (0.9% Sodium chloride)	MB PHARMA s.r.o	Phase 1 / Phase 2a	NCT06319235
Phage therapy for the prevention and treatment of wound infections in burned patients	**	Wound Infection (burns infected or susceptible to S. aureus, P. aeruginosa, K. pneumoniae)	Biological: bacteriophage cocktail spray (Phage Cocktail‐SPK) Drug: xeroform	Precisio Biotix Therapeutics, Inc.	Phase 1	NCT04323475
Mycobacteriophage treatment of nontuberculosis mycobacteria	Enrolling by invitation	CF, NTM lung disease, pulmonary mycobacterial infections	Mycobacteriophage	National Jewish Health	*	NCT06262282
Phageinlyon clinic cohort study: a descriptive study of severe infections treated with phage therapy at the HCL	Recruiting	Severe infection	Description of severe infection	Hospices Civils de Lyon	*	NCT06185920
Clinical study of phage therapy for chronic constipation efficacy and safety	Recruiting	Pib‐specific phage, intractable constipation	Phage	Shanghai 10th people's hospital	*	NCT05973721
Treatment chronic UTI post‐kidney transplant	Recruiting	UTIs, transplant‐related disorder	Biological: phage	Shahid beheshti university of medical sciences	Phase3	NCT05967130
Bacteriophage therapy TP‐102 in patients with diabetic foot infection	Recruiting	Diabetic foot infection	Biological: TP‐102 Other: placebo	Technophage, SA	Phase 2	NCT05948592
Ph 1/2 study evaluating safety and tolerability of inhaled AP‐PA02 in subjects with chronic *P. aeruginosa* lung infections and cystic fibrosis	Completed	CF, *P. aeruginosa*, *Pseudomonas*, lung infection, lung infection pseudomonal	Biological: AP‐PA02 Other: placebo	Armata Pharmaceuticals, Inc.	Phase 1/2	NCT04596319
Phage therapy for the treatment of UTI	Active_not_recruiting	Recurrent UTI	Biological: phage therapy (HP3, HP3.1, ES19)	Unity Health Toronto	Phase 1/2	NCT05537519
Bacteriophage therapy of difficult‐to‐treat infections	Completed	Bacterial infections	Biological: bacteriophage therapy	Queen Astrid Military Hospital	*	NCT05498363
A study of LBP‐EC01 in the treatment of acute uncomplicated UTI caused by drug‐resistant *E. coli* (eliminate trial)	Recruiting	UTIs	LBP‐EC01 (0.1× IV dose, 0.01× IV dose, IV Infusion), Placebo, TMP/SMX	Locus Biosciences	Phase 2	NCT05488340
A phase 1b/2 trial of the safety and microbiological activity of bacteriophage therapy in CF Subjects Colonized With *P. aeruginosa*	Completed	Bacterial disease carrier, CF	Other: placebo Biological: WRAIR‐PAM‐CF1	NIAID	Phase 1b/2	NCT05453578
Mayo clinic phage program biobank	Enrolling by invitation	Bacteriophage therapy	Observational registry; collection of biospecimens (blood, synovial fluid)	Mayo clinic	*	NCT05314426
Bacteriophage therapy in patients with diabetic foot osteomyelitis	Recruiting	Osteomyelitis, diabetic foot osteomyelitis	Biological: phage therapy Other: placebo	Adaptive phage therapeutics, inc.	Phase 2a	NCT05177107
Nebulized bacteriophage therapy in CF patients with chronic *P. aeruginosa* pulmonary infection	Active_not_recruiting	Chronic *P. aeruginosa* infection, CF	Drug: bx004‐a Drug: placebo	Biomx, inc.	Phase 1/2	NCT05010577
Phage therapy for the prevention and treatment of pressure ulcers	**	Pressure ulcer	Combination product: bacteriophage‐loaded microcapsule spray Combination product: placebo Procedure: standard of care	Precisio Biotix Therapeutics, Inc.	Phase 1/2	NCT04815798
Personalized phage treatment in COVID‐19 patients with bacterial coinfections microbials for pneumonia or bacteremia/septicemia	No longer available	COVID‐19, bacteremia, septicemia, *A. baumannii* Infection, *P. aeruginosa* Infection, *S. aureus* Infection	Phage therapy	Adaptive Phage Therapeutics, Inc.	Not applicable (Expanded Access)	NCT04636554
Bacteriophage therapy TP‐102 in diabetic foot ulcers	Completed	Diabetic foot ulcer; *P. aeruginosa*; *S. aureus*; *Acinetobacter* infections	TP‐102	Technophage, SA	Phase 1/2a	NCT04803708
Antibacterial treatment against diarrhea in oral rehydration solution	Terminated	Diarrhea	T4 phage cocktail test Commercial T4 phage cocktail Standard ORS	SPN	Not Applicable	NCT00937274
Standard treatment associated with phage therapy versus placebo for diabetic foot ulcers infected by *S. aureus*	**	Diabetic foot Staphylococcal infections	Drug: topical anti‐*Staphylococcus* bacteriophage therapy Drug: topical placebo corresponding to anti‐*Staphylococcus* bacteriophage therapy	Centre Hospitalier Universitaire de Nīmes	Phase 1/2	NCT02664740
CF bacteriophage study at yale (CYPHY)	Completed	CF	Drug: standard dose YPT‐01 Other: placebo	Yale university	Phase 1/2	NCT04684641
Bacteriophage therapy in tonsillitis	Active_not_recruiting	Acute tonsillitis	Nebulizer inhalation irrigation of tonsil mucous membranes with a bacteriophage	Tashkent State Medical University (Tashkent Pediatric Medical Institute), Uzbekistan	Phase3	NCT04682964
Phage safety cohort study	Recruiting	PJI, severe infection	Adverse event after injection of phages	Hospices Civils de Lyon	*	NCT04650607
Experimental phage therapy of bacterial infections	**	Bacterial infections	Other: bacteriophage preparation	Institute of Immunology and Experimental Therapy of the Polish Academy of Sciences	Not Applicable	NCT00945087
Study to evaluate the safety, phage kinetics, and efficacy of inhaled AP‐PA02 in subjects with non‐CF bronchiectasis and chronic pulmonary *P. aeruginosa* infection (tailwind)	Completed	Non‐CF bronchiectasis, *P. aeruginosa*, Lung Infection	Biological: AP‐PA02| Other: placebo	Armata Pharmaceuticals, Inc.	Phase 2	NCT05616221
Evaluation of phage therapy for the treatment of *E. coli* and *P. aeruginosa* wound infections in burned patients	**	Wound Infection	Drug: *E. coli* phages cocktail Drug: standard of care: silver sulfadiazine Drug: *P. aeruginosa*, phages cocktail	Phaxiam Therapeutics	Phase 1/2	NCT02116010
Bacteriophage therapy in patients with UTIs	Terminated	UTI bacterial	Bacteriophage therapy	Adaptive Phage Therapeutics, Inc.	Phase 1/2	NCT04287478
Bacteriophages for treating UTIs in patients undergoing transurethral resection of the prostate	Completed	Intravesical bacteriophage treatment for UTIs	Biological: PYO phage Drug: antibiotics Other: sterile bacteriology media	Balgrist University Hospital	Phase 2/3	NCT03140085
**Endolysin**						
First‐in‐man single‐dose and multiple dose study to evaluate the safety, tolerability and efficacy of HY‐133 (HY‐133)	Recruiting	Nasal colonization with *S. aureus*	Drug: HY‐133 (recombinant chimeric bacteriophage endolysin), placebo	University Hospital Tuebingen	Phase 1	NCT06290557
A Study to evaluate the safety, pharmacokinetics and pharmacodynamics of N‐Rephasin® SAL200 in healthy male volunteers	Completed	Healthy volunteers anti‐bacterial agents methicillin‐resistant *S. aureus*	Biological: N‐Rephasin® SAL200 Other: INT200‐Placebo	Intron Biotechnology, Inc.	Phase I	NCT01855048

Abbreviations: AP‐PA02, inhaled bacteriophage therapy; CF, cystic fibrosis; DAIR, debridement, antibiotics, and implant retention; E. coli, Escherichia coli; HCL, Hospices Civils de Lyon; IV, Intravenous; MSSA, methicillin‐sensitive Staphylococcus aureus; NaCl, sodium chloride; NCT, National Clinical Trial; NIAID, National Institute of Allergy and Infectious Diseases; NTM, nontuberculosis mycobacteria; ORS, Oral Rehydration Solution; PJI, prosthetic joint infection; S. aureus, Staphylococcus aureus; SPN, Société des Produits Nestlé; TMP/SMX, trimethoprim/sulfamethoxazole; UTI, urinary tract infection; VAP, ventilator‐associated pneumonia.

* Denotes observational study for adverse events related to bacteriophage therapy.

** Study has passed its completion date and status has not been verified in more than 2 years. Data are extracted from ClinicalTrials.gov (ClinicalTrials.gov).

### 6.2 Approved Uses of Phage Therapy

Phage therapy has been widely adopted in countries like Poland, Georgia, and Russia, but with significant regulatory differences. Poland, as an EU member, follows stringent EU regulations, treating phage therapy as an experimental treatment under “compassionate use” [195]. This approach allows patients access to phage therapy even though it is not fully approved. In contrast, Georgia and Russia have more lenient approaches. In Georgia, personalized phage preparations are manufactured under government approval [196], while Russia permits the sale of ready‐made phage cocktails but prohibits personalized phage products [197]. Western European countries like Belgium have introduced magistral preparations, allowing pharmacists to custom‐make phage treatments, setting a potential model for broader EU regulation [198]. The European Pharmacopoeia is working toward standardizing phage products, aiming to bring consistency and legal clarity across the EU in the coming years [195].

### 6.3 Real‐World Case Studies of Phage and Endolysin Therapy in Compassionate Use Scenarios

**FIGURE 1:**
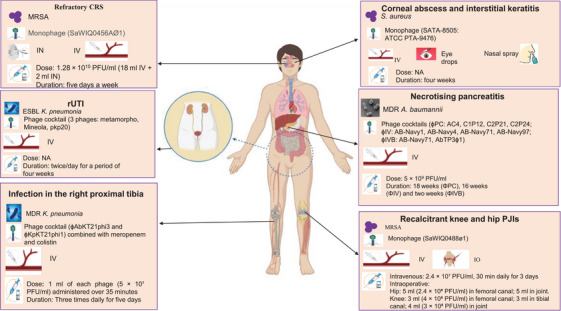
Overview of some human case studies on phage therapy for drug‐resistant pathogens. This figure presents various therapeutic applications of phage therapy for treating multidrug‐resistant (MDR) infections. The cases include: *refractory chronic rhinosinusitis (CRS)*: treated with a monophage (SaWIQ0456A01) targeting MRSA. Administered intravenously (IV) and intranasally (IN) at a dose of 1.28 × 10¹⁰ PFU/mL, 5 days per week. *Recurrent urinary tract infection (rUTI)*: due to ESBL‐producing K. pneumoniae, managed with a phage cocktail (metamorpho, Mineola, pkp20) administered intravenously twice daily for 4 weeks. *Corneal abscess and interstitial keratitis*: caused by *S. aureus*, treated with a monophage (SATA‐8505: ATCC PTA‐9476) applied as eye drops and nasal spray over 4 weeks. *Necrotizing pancreatitis*: associated with MDR A. baumannii, treated with a combination of phage cocktails (ϕPC, ϕIV, ϕVB), administered intravenously at a dose of 5 × 10⁸ PFU/mL over varying durations: 18 weeks (ϕPC), 16 weeks (ϕIV), and 2 weeks (ϕVB). *Infection in right proximal tibia*: due to MDR *K. pneumoniae*, addressed with a phage cocktail (ϕAbKT21phi3 and ϕKpKT21phi1) combined with meropenem and colistin, delivered intravenously three times daily over 5 days. *Recalcitrant knee and hip periprosthetic joint infections (PJIs)*: Treated with a monophage (SaWIQ0458a1) against MRSA. Administered intravenously (2.4 × 10⁷ PFU/mL, daily for 3 days) and intraoperatively (IO) with specified doses for hip and knee injections. Each case demonstrates the therapeutic potential of phages as a targeted treatment for resistant infections, showcasing diverse routes of administration and dosage regimens. Figure created using BioRender and Microsoft PowerPoint.

The first successful intravenous bacteriophage therapy in the United States occurred in March 2016 for a multiantibiotic‐resistant A. baumannii infection at the University of California San Diego [199]. This case renewed interest in phage therapy, leading to public presentations and an United States Food and Drug Administration (US FDA) meeting in August 2021 on its science and regulation [200]. The US FDA has granted emergency use authorization for compassionate use of phage therapy on a case‐by‐case basis, allowing patients with no other options to receive investigational products [199]. Optimal candidates for compassionate phage therapy include patients with chronic infections, such as cystic fibrosis, who often experience antibiotic resistance. A 2005 study evaluated 153 patients with MDR infections, showing favorable tolerance and outcomes [201].The Antibacterial Resistance Leadership Group reviewed 63 single‐patient investigational new drug cases, finding favorable outcomes in 51 cases [202]. Additionally, a retrospective analysis indicated promising results for phage therapy in chronic bacterial infections unresponsive to antibiotics [201]. Encouraging clinical outcomes have been reported in single‐patient phage therapy cases, including a study at UC San Diego where seven of 10 patients with MDR infections improved [203] and another demonstrating rapid improvement in a M. chelonae infection after unsuccessful antibiotic treatment [204]. Phage therapy is being explored for MDR infections in transplant recipients and secondary bacterial infections in COVID‐19 patients [205, 206]. A review reported treatment of 70 patients with various infections, including cutaneous infections (3%), prostatitis (9%), and urinary tract infections (9%) [207]. Administration regimens vary, with no standardized schedule recommended [202]. Chronic urinary tract infections have been treated with oral phage preparations, while ventilator‐associated pneumonia cases have successfully used both intravenous and nebulized phage therapies [208]. Despite promising outcomes, limitations include variability in administration protocols and efficacy assessment challenges due to heterogeneous bacterial populations [209]. Efforts are underway to standardize phage therapy protocols, as seen at the Hirszfeld Institute of Immunology and Experimental Therapy (HIIET) in Poland [201]. Continued clinical trials are needed to validate phage therapy's efficacy across diverse populations. Early evidence suggests phage therapy, particularly when combined with antibiotics, can reduce infection severity and resistance [202]. One notable study by Pal and colleagues [132] reviewed human case studies of phage therapy targeting drug‐resistant ESKAPEE pathogens, emphasizing the therapeutic potential of phage‐based approaches against MDR bacteria. Most patients experienced significant improvement or full recovery, especially when conventional antibiotics failed. Phage therapy was used effectively to treat various infections, including recurrent UTIs, CBP, prosthetic joint infections, and more (Figure 1). It was administered via multiple routes, such as intravenous, intranasal, and topical, demonstrating flexibility in treating different infection sites. In many cases, phage therapy was combined with antibiotics, leading to faster recovery and better outcomes. Patients tolerated the treatment well, with minimal adverse effects. Long‐term follow‐up showed low recurrence rates, suggesting the lasting effectiveness of phage therapy. Overall, these studies underscore phage therapy's potential as a valuable alternative for treating MDR infections when no other options remain.

## 7 Challenges in Translating Phage and Endolysin Therapy to the Clinic

Despite the growing potential of phages as substitutes or complements to antibiotics in treating bacterial infections, studies underscore the obstacles that must be overcome to make this innovative approach more accessible in clinical settings.

### 7.1 Manufacturing and Production Issues

Maintaining stability during application is a significant challenge in the production of bacteriophages. Factors including temperature, pH, ion concentration, the presence of seafood matrices, and UV exposure duration have been identified as influencing the stability of bacteriophages. Temperature plays a crucial role in influencing the activity of bacteriophages. Exposure to suboptimal temperatures prolongs the latent period of bacteriophages, resulting in limited participation in proliferation [210, 211, 212]. The temperature tolerance of bacteriophages varies substantially based on the climatic conditions of their source location. Likewise, the pH and ion concentration ranges tolerated by bacteriophages generally mirror the conditions found in their host environment. Exposure to UV radiation can modify the genetic material of bacteriophages or degrade their proteins, leading to the creation of photoproducts such as cyclobutene pyrimidine dimers. The unpredictability of these factors in real‐world applications poses a significant barrier to the broad adoption of bacteriophages [213, 214]. Ensuring comprehensive security in phage applications poses an additional challenge in the manufacturing process. Ensuring comprehensive security in phage applications poses an additional challenge in the manufacturing process. A notable instance is phage EC10, which can transfer the *bla* resistance gene between different strains of *E. coli*, such as from NBRC 12713 KEN1 to C600RK2, HB101, NBRC 12713, and W3110 [2]. Optimizing the dosage of bacteriophage administration poses a significant challenge. Once bacteriophages enter the body and interact with various organs, their efficiency and survival rates should mirror those observed in laboratory tests. In cases where a local infection progresses to a systemic one, the phage candidate must survive sufficiently to exert its effects at the infection site. When administered via the intestine, the candidate phage must be able to survive in the bloodstream. A key challenge lies in assessing the antibacterial efficacy of bacteriophages. Maintaining the quality of selected phages is crucial for clinical use. The concentration of phages exhibits a linear correlation with their ability to inhibit bacterial growth. Certain phages remain effective at concentrations as low as 10^5^/mL. Conversely, some studies have involved administering phage lysates intravenously at concentrations of up to 10^10^ pfu/mL. Due to the limited availability of clinical trial data, it is challenging to monitor serum phage concentrations and establish the effective titer required for therapeutic efficacy [215]. There has been a growing consumer preference for natural and organic products over those treated with antibiotics or chemicals in recent years. This shift in consumer preference has led to a rise in the use of phages in the food industry. Despite the potential advantages of phages, public awareness remains limited, which substantially impedes their broader application in the food sector. Consequently, prioritizing public education and raising awareness among both consumers and food industry professionals about the safety and effectiveness of phage applications is crucial for promoting their more widespread adoption [2]. The development of resistance to bacteriophages presents a major obstacle to their practical application, necessitating strategies to prevent the emergence of resistant bacterial strains and counteract their defense mechanisms. Bacterial resistance to phage infection can be classified into five distinct categories based on the underlying mechanisms: interference with phage attachment, inhibition of phage DNA entry, disruption of DNA replication, abortive infection processes, and obstruction of phage assembly and release [216].

### 7.2 Regulatory Approval Hurdles for Phage Therapy

In the era of the former Soviet Union, bacteriophages were mass‐produced and available for use, with the first clinical trials dating back to the 1920s. In the era of the former Soviet Union, bacteriophages were mass‐produced and available for use, with the first clinical trials dating back to the 1920s. Currently, establishing regulatory guidelines remains challenging due to the need for a controlled manufacturing process in compliance with GMP for phage production. The complex nature of phages makes them less suitable for GMP compliance, as their production is time consuming and costly for pharmaceutical companies, further complicated by limited patentability [215]. Regulatory models tailored for non‐GMP phages should be considered. In the United States, phages are used to treat patients via the US FDA's Expanded Access pathway, which includes emergency investigational new drug applications. This process requires documentation clearly stating that antibiotic therapy has failed. A significant challenge in phage therapy is the scarcity of clinical trial data needed to support phages as medicinal products under GMP and regulatory standards. Over the past few years, numerous companies and researchers worldwide have introduced innovative ideas that are helping to advance phage therapy toward market viability. In the future, it may become possible to obtain personalized medicine licenses for unique regimens that combine phages with antibiotics, engineered phages, or natural generic phages [217].

### 7.3 Intellectual Property and Commercial Development Concerns

By adopting continuous production methods, the issues of low yield, variable quality, and elevated costs inherent in batch production of bacteriophages can be effectively mitigated. Continuous phage production involves a steady supply of fresh medium and simultaneous removal of spent medium containing bacteria and phages. Prolonged cocultivation in single‐stage continuous reactors, such as chemostats or turbidostats, can result in genetic mutations, rendering them less ideal for large‐scale phage production due to these limitations. The development of two‐stage continuous reactors, known as cellstats, aims to minimize the occurrence of mutations. These systems comprise two tanks of varying capacities that are continuously stirred, along with a storage tank for the final product [218, 219]. In the larger tank, host bacteria are cultivated, with their growth regulated by the addition of fresh medium to maintain an exponential growth phase that facilitates phage infection. By culturing host bacteria separately in the initial fermenter, the emergence of phage‐resistant bacteria is substantially diminished. Once prepared, the host bacteria culture is moved to the second reactor where infection occurs. For successful infection, the rate at which host bacteria are introduced must align with the phage infection rate, as mismatching these rates can result in phages exiting the fermenter without infecting the bacteria [220, 221]. A significant barrier to the broader implementation of phage therapy lies in the economic landscape. Pharmaceutical companies can attain success by scaling up phage research into profitable ventures. Several concerns are linked to investing in bacteriophage therapy at the pharmaceutical level. A major challenge is the limited potential for patenting phages. Since phages are classified as natural products, while they can be lifesaving in cocktail formulations, their patents are often considered weak. In these situations, genetically engineered phages with novel modifications that enhance antibacterial activity may be eligible for patent protection. Natural phages used in research and clinical trials pose a challenge for financial investment due to their fragile patent status, which complicates achieving regulatory standards [222, 223].

### 7.4 Patient‐Specific Phage Cocktails versus Standardized Treatment Options

The dynamics between bacteria and bacteriophages at infection sites remain poorly comprehended due to their intricate nature. Replicating bacterial populations within biofilms, which thrive in competitive polymicrobial environments, poses significant challenges in laboratory settings during the preclinical assessment of phage‐based treatments. Strategies for dosing and delivery that are developed through in vitro research often fail to yield successful outcomes when applied in vivo [224]. The formulation and delivery method of a product are crucial elements tied to its target profile, which includes its intended use and indication—specifically, treating infections caused by particular bacterial strains that are susceptible to either a single phage or a phage cocktail. Developing formulations for various phages is a key component of chemistry, manufacturing, and controls activities, which focus on enhancing the shelf life of phage‐based drug substances. Phage cocktails pose difficulties in precisely determining the effectiveness of each individual phage within the final formulation. Using a common host for plating and PCR to assess plaque identities can offer partial validation of the potency of the various phages within these cocktails [224]. Standardizing phage therapy preparations poses significant challenges. There is ongoing uncertainty regarding the precise dosage definition. The effectiveness of phage therapy is heavily influenced by both the method of delivery and the dosage, complicating its clinical use. Due to their composition of proteins and nucleic acids, bacteriophages are prone to degradation upon interaction with human metabolic processes, particularly in the stomach and liver, as well as when facing the immune response in animals. Pharmacokinetic research revealed that a portion of bacteriophage infusions persisted for 36 h posttreatment, though their potency was diminished by dilution in bodily fluids [225]. Oral delivery emerged as the most appropriate method for both humans and animals, offering ease, comfort, and minimal immune response compared with alternative administration techniques. As bacteriophages traverse the stomach, intestine, and intestinal lining during oral administration, the gastrointestinal system acts as a major obstacle to their entry into tissues. Moreover, the mammalian circulatory system efficiently clears bacteriophages from the bloodstream, making it challenging to sustain the necessary concentrations required to eliminate target bacteria [225].

## 8 Future Directions and Innovations

### 8.1 Genetic Engineering of Phages to Improve Efficacy

**FIGURE 2:**
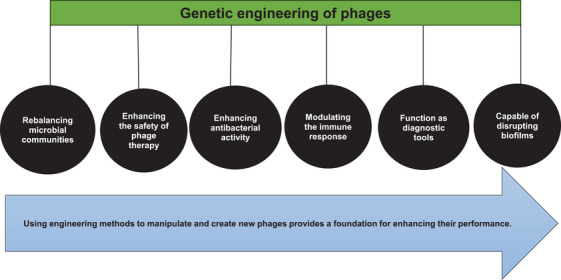
Key functional properties enhanced through genetic engineering of phages. This schematic illustrates the primary capabilities that can be optimized by manipulating natural bacteriophages through bioengineering techniques. These improvements include rebalancing microbial communities, increasing the safety and antibacterial efficacy of phage therapy, modulating host immune responses, enabling diagnostic functionality, and disrupting biofilms. Such advancements collectively contribute to the development of more effective and targeted phage‐based therapeutic agents. Figure created using Microsoft PowerPoint.

Genetically engineered phages and endolysins have shown promise in overcoming the limitations of traditional phages and antibiotics, particularly against bacteria that are resistant to multiple drugs, XDR, or form biofilms [15]. The creation and use of modified phages require collaboration across multiple fields, including biotechnology, microbiology, medicine, genetics, and biology (Figure 2). This complex process involves various genetic engineering methods, such as CRISPR–Cas genome editing, homologous recombination, yeast‐based assembly of phage DNA, cell‐free systems for transcription and translation, electroporation for DNA introduction, genetic recombination in living organisms, and laboratory manipulation of phage genetic material. These techniques are crucial for producing genetically modified phages and endolysins [226]. It is well understood that disturbances in the body's microbial communities can have harmful effects on human health. On the other hand, engineered phages provide a viable method for rebalancing microbial communities [227, 228, 229, 230]. Bioengineering overcomes the limitations of natural phages under difficult conditions. Enhancing the safety of phage therapy involves creating phages that prevent the release of bacterial toxins during cell lysis [231]. By modulating the immune response and genetically modifying phages through the deletion of holin–endolysin system genes, it is possible to create nonlytic phage variants that effectively target bacteria while minimizing endotoxin release and reducing harmful inflammatory reactions [232, 233, 234]. Engineered phages can function as diagnostic tools. For example, integrating luciferases into phage DNA allows for the rapid and precise detection of bacteria. Bioengineering techniques have been used to modify phage tail‐fiber and tail genes, broadening the range of proteins they encode to specifically target bacteria at the species level [226]. This genetic modification enables phages to infect and distinguish new bacterial hosts, transcending species limitations. Genetically engineered phages are capable of disrupting biofilms that are resistant to antimicrobial treatments. Although some phages do not naturally produce depolymerase, genetic engineering can be used to create phages that do. These modified phages, which can degrade biofilms and polysaccharide capsules, have shown efficacy in fighting bacterial biofilms [226].

### 8.2 Development of Synthetic Phages and Endolysins

AIEgens‐PAP is a synthetic phage that represents a novel type of antimicrobial biological conjugate created by combining AIEgens, a photosensitizer with photodynamic inactivation properties, with PAP. Recombinant AIEgens specifically targeted *P. aeruginosa*, generating reactive oxygen species and exhibiting enhanced bactericidal activity [235]. In 2022, researchers demonstrated a versatile cell‐free platform designed to advance the production, proteomic analysis, and transient engineering of bacteriophages. This nongenomic approach to phage engineering exhibits therapeutic potential against MDR bacteria by enabling functionality for only a single replication cycle [236]. Recent research introduced engineered anti‐CRISPR phages containing anti‐CRISPR (Acr) genes such as AcrIF1, AcrIF2, and AcrIF3. These genes were incorporated into phage DMS3/DMS3m to inhibit infection and replication of *P. aeruginosa*. This innovative approach demonstrated remarkable antibacterial efficacy and high safety in combating MDR *P. aeruginosa* isolates [237]. Another study involved the administration of a specially engineered three‐phage cocktail to a 15‐year‐old cystic fibrosis patient suffering from a disseminated drug‐resistant *Mycobacterium abscessus* infection. Intravenous treatment with a lytic phage successfully eradicated the infectious *M. abscessus*, was well tolerated by the patient, and led to significant clinical improvements. These included enhanced liver function, wound healing, and notable reductions in infected skin nodules [238].

For an engineered endolysin to be effective in medical applications, it must be capable of eradicating bacteria in complex environments such as mucosal membranes, animal tissues, bodily fluids, and blood. In addition to improving its bactericidal properties, alterations to phage lytic enzymes can enhance various attributes, including stability, activity range, solubility, and longevity within infected hosts. Although phage lytic enzymes are expected to trigger an immune response due to their protein composition and the induction of bacterial lysis, it is important to note that antibodies targeting these enzymes have not shown significant adverse effects [181]. These modified lytic enzymes can be delivered through various routes to treat infections, such as orally, intratracheally, intramammarily, intramuscularly, intravenously, intranasally, topically, subcutaneously, and intraperitoneally [239]. Several modified enzymes and endolysins have been studied for their enhanced functional properties. The improvements in endolysin or enzyme characteristics have resulted from chemical modifications that involve fusing them with different substances like silver nanoparticles, AIEgens, pheophorbide A, cellulose membranes, and indium tin oxide. These novel compounds have proven effective against infections and employ various mechanisms to combat bacteria, which complicates the development of resistance mechanisms [240]. For instance, combining phage T4 lysozyme with pesticin has resulted in improved penetration and a greater ability for the compound to reach PG [241]. PM‐477 is a compound developed through domain shuffling that shows increased bactericidal effectiveness and biofilm removal abilities while maintaining the vaginal microbiome's integrity [242]. Two original lysins have been combined to create chimeolysins ClyR and ClyF, which exhibit resistance to heat‐inactivated human serum as well as mouse serum, human serum, and rabbit serum. They also demonstrate robust lytic activity and a broader range of streptococcal hosts [85, 243, 244]. Additionally, a new compound called LNT113 has been created by fusing cecropin A with EC340 endolysin, resulting in improved outer membrane permeability, enhanced activity, and synergistic effects with antibiotics [245]. Furthermore, Artilysin, which is a fusion of an additional endolysin and a peptide, has shown enhanced antimicrobial effectiveness and improved penetration capabilities [246]. A newly developed endolysin named CP25L has been successfully produced and delivered into the gastrointestinal tract using a genetically engineered probiotic strain [247].

### 8.3 Use of Phage Therapy in Combination with Traditional Antibiotics

**FIGURE 3:**
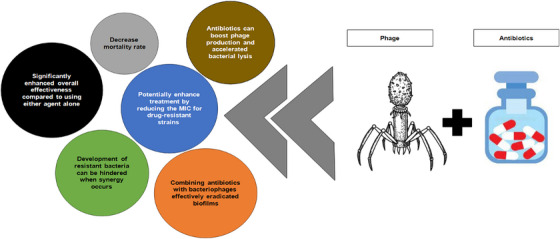
Synergistic effects of combining bacteriophages and antibiotics in antimicrobial therapy. The diagram illustrates key advantages of phage‐antibiotic synergy, including increased treatment effectiveness, reduced minimum inhibitory concentration (MIC) for drug‐resistant strains, enhanced biofilm eradication, and inhibition of resistant bacteria development. Antibiotics can also promote phage production and accelerate bacterial lysis. These interactions suggest promising potential for combined therapies, particularly in overcoming antibiotic resistance. Figure created using Microsoft PowerPoint.

The synergy between bacteriophages and antibiotics results in a significantly enhanced overall effectiveness compared with using either agent alone (Figure 3). Studies have shown that sublethal concentrations of β‐lactam and quinolone antibiotics can boost phage production, leading to higher PFU counts. The observed increase in phage production and accelerated bacterial lysis may be attributed to the enhanced filamentation of bacterial cells when exposed to antibiotics [248]. This synergy holds potential for clinical applications in improving bacterial treatments, but it requires additional research to clarify several aspects. One unresolved question is whether bacteriophages should be used alone, in combination with other phages, or alongside antibiotics to maximize bacterial cell lysis. Another open question concerns the optimal timing of phage and antibiotic administration—whether they should be given together or one after the other [248]. Laboratory studies on the interaction between phages and antibiotics have revealed that combining antimicrobials with bacteriophages can amplify their effectiveness and extend their reach to bacteria that were initially resistant. Research on the bacteriophage ΦHP3 combined with various antimicrobials against extraintestinal *E. coli* strains indicates that synergy depends mainly on the mechanism of action of the antibiotic and the concentrations used, as observed with ciprofloxacin. The timing of administration also plays a role in influencing the synergistic effect observed between phages and antibiotics. While human trials are lacking, animal studies have provided evidence supporting the simultaneous use of bacteriophages and antimicrobials [249, 250]. A study explored the effectiveness of combining ciprofloxacin with bacteriophage therapy for treating *P. aeruginosa* endocarditis in rats. Approximately 64% of the rats receiving the combined treatment showed negative vegetation cultures just 6 h posttreatment [251]. Synergistic outcomes were observed when bacteriophage therapy was added to standard enrofloxacin treatment. The combination group experienced zero mortality, contrasting with a 3% mortality rate in the enrofloxacin group and 15% in the bacteriophage group [252]. An in vitro MRSA model was used to assess the effectiveness of combining fosfomycin, vancomycin, oxacillin, and ciprofloxacin with bacteriophages. Checkerboard analysis validated the effectiveness of these combinations, showing a decrease in the MIC for all antibiotics tested, including oxacillin. Additional analysis using a *G. mellonella* model showed that combining oxacillin with bacteriophages effectively eradicated biofilms, especially when the antibiotic was given before the phage solution. Phages have been shown to potentially enhance treatment by reducing the MIC for drug‐resistant strains under specific conditions [253]. Combining phages with antibiotics can result in either synergistic or antagonistic effects, depending on how the paired antibiotic class inhibits bacterial growth. The development of resistant bacteria can be hindered when synergy occurs between phages and antibiotics [249]. Western companies developing phage cocktails often overlook key virological characteristics of phages, including target specificity and antagonistic coevolution. Consequently, the underwhelming performance of standardized phage cocktails in recent clinical trials is unsurprising, especially when compared with the promising outcomes from case studies using phages as adjunctive treatments or employing preadapted or bioengineered phages [248].

### 8.4 Personalized Phage Therapy Approaches

Personalized phage therapy involves selecting phages from either a phage bank or the environment and, if necessary, adapting them through in vitro selection to enhance their ability to infect bacteria isolated from the patient's infection site. Certain phage therapy centers establish and maintain extensive therapeutic phage banks, continually updating them with novel phages to expand and adapt the host range in response to evolving bacterial populations. The sustainability of personalized phage therapy is enhanced because it specifically targets the infecting bacterium, thereby reducing the pressure on bacteria to develop resistance to phages [254]. Nevertheless, personalized approaches are more intricate and logistically challenging compared with universal treatments, involving the global distribution of bacterial strains and their corresponding phages. Additionally, precision medicine principles generally do not align with typical drug development and licensing processes, which are lengthy and costly, requiring substantial time and financial investment for each phage in the bank. Western regulatory frameworks have begun to accommodate precision and personalized phage therapy methods, incorporating naturally occurring, engineered, and synthetic phages [254]. Additionally, various researchers and clinicians support the use of phage therapy as personalized medicine, citing benefits such as minimizing the development of phage resistance in bacterial populations. Initially, employing phages in a moderate and personalized manner can help mitigate the intense evolutionary pressures on bacterial populations that often result from high doses of other antimicrobial treatments. Additionally, recent publications highlight that the potential for immune sensitization to phages and their subsequent elimination upon repeated use should not be overlooked. Furthermore, customized phage approaches, like the recently approved “magistral preparation” in Belgium, offer a cost‐effective and expedited alternative to traditional drug approval processes. To sustain the effectiveness of phage therapies, careful implementation is crucial, learning from past errors with other antimicrobials. Currently, personalized phage therapy appears to be a promising approach to achieve this goal [255]. A case report details the treatment of a patient suffering from a pandrug‐resistant *P. aeruginosa* spinal abscess, involving surgical intervention combined with a tailored phage therapy regimen supplemented by antibiotics. Since the *P. aeruginosa* strain proved resistant to phages from private companies, a novel European academic collaboration was established to develop, manufacture, and timely administer a customized phage cocktail for the patient. Following two surgical procedures, despite ongoing bacterial presence and the emergence of small colony variants, the patient recovered through the use of purified phages administered via local and intravenous injections as supplementary therapy [256].

## 9 Conclusion

The emergence of antibiotic‐resistant bacteria is a predictable aspect of human health, posing significant challenges for both patients and medical professionals in clinical settings. As a result, researchers have long sought alternatives to antibiotics, with bacteriophages being recognized as promising biological agents due to their efficacy. Currently, the isolation and application of phage enzymes and endolysins are proving effective against antibiotic‐resistant bacteria, including those classified as MDR, XDR, and PDR. Studies conducted in both animals and humans indicate that phages offer a promising avenue for treating complex bacterial infections in clinical environments. However, several challenges hinder the optimal use of phages, both individually and in combination, prompting ongoing research to develop effective solutions. Further clinical research is necessary to elucidate the precise interactions between phages and their hosts, which will facilitate the development of more precise and effective phage formulations. In the foreseeable future, it is plausible that engineered phages, engineered endolysins, and enhanced phage cocktails will become common treatments for antibiotic‐resistant infections, including those resistant to last‐resort antibiotics.

## Author Contributions

S.S., S.N., and M.H.B. designed, supervised the project, and edited the final manuscript. M.T.M., S.M., and R.S. wrote different parts the manuscript. All authors have read and approved the final manuscript.

## Ethics Statement

The authors have nothing to report.

## Conflicts of Interest

The authors declare that the research was conducted in the absence of any commercial or financial relationships that could be construed as a potential conflict of interest.

## Data Availability

The authors have nothing to report.
